# The mother-to-child transmission of HIV-1 and profile of viral reservoirs in pediatric population: A systematic review with meta-analysis of the Cameroonian studies

**DOI:** 10.1371/journal.pone.0278670

**Published:** 2023-01-17

**Authors:** Aude Christelle Ka’e, Alex Durand Nka, Bouba Yagai, Irénée Domkam Kammogne, Ezechiel Ngoufack Jagni Semengue, Aubin Joseph Nanfack, Celine Nkenfou, Michel Carlos Tommo Tchouaket, Desire Takou, Samuel Martin Sosso, Nadine Fainguem, Aissatou Abba, Willy Pabo, Nelly Kamgaing, Edith Temgoua, Boris Tchounga, Patrice Tchendjou, Suzie Tetang, Anne Esther Njom Nlend, Francesca Ceccherini-Silberstein, Maria Mercedes Santoro, Joseph Fokam

**Affiliations:** 1 Chantal Biya International Reference Centre for Research on HIV/AIDS Prevention and Management (CIRCB), Yaounde, Cameroon; 2 University of Rome “Tor Vergata” (UTV-Rome), Rome, Italy; 3 Concern Worldwide, Niamey, Niger; 4 Higher Teachers Training College, University of Yaounde I, Yaounde, Cameroon; 5 Catholic University of Central Africa (UCAC), Yaounde, Cameroon; 6 University of Buea, Buea, Cameroon; 7 National AIDS Control Committee (CNLS), Yaounde, Cameroun; 8 Elisabeth Glaser Pediatric AIDS Foundation (EGPAF), Douala, Cameroon; 9 National Social Welfare Hospital (CHE), Yaounde, Cameroon; 10 Higher Institute of Medical Technology (ISTM), Yaounde, Cameroon; The Technical University of Kenya, KENYA

## Abstract

**Background:**

The mother-to-child transmission of HIV-1 (MTCT) remains on the major route of HIV-transmission among pediatric populations in Africa. Though a prevention of MTCT (PMTCT) high-priority country, data on the MTCT burdens in Cameroon remains fragmented.

**Objective:**

We sought to assess the pooled MTCT rate, its risk-factors, and to characterize viral reservoirs of infected-children in Cameroon.

**Methods:**

All relevant observational cohort and cross-sectional studies conducted in Cameroon were searched from PubMed, African Journals Online, Google scholar, ScienceDirect and academic medical education databases. Heterogeneity and publication bias were respectively assessed by the I^2^ statistic and the Egger/funnel plot test. Meta-analysis was performed using the random effects model. MTCT rate >5% was considered as “high”. This review was registered in the Prospero database, CRD42021224497.

**Results:**

We included a total of 29 studies and analyzed 46 684 children born from HIV-positive mothers. The overall rate of MTCT was 7.00% (95% CI = 6.07–8.51). According to regions, the highest burden was in Adamaoua-region (17.51% [95% CI:14.21–21.07]) with only one study found. PMTCT option-B+ resulted in about 25% reduction of MTCT (8.97% [95% CI: 8.71–9.24] without option-B+ versus 2.88% [95% CI: 5.03–9.34] with option-B+). Regarding risk-factors, MTCT was significantly associated with the absence of PMTCT-interventions both in children (OR:5.40 [95% CI: 2.58–11.27]) and mothers (OR: 3.59 [95% CI: 2.15–5.99]). Regarding viral reservoirs, a pro-viral DNA mean of 3.34±1.05 log_10_/mL was observed among 5/57 children and archived HIV drug resistance mutations were identified in pro-viral DNA marker among 21/79 infected-children.

**Conclusion:**

In spite of the dropdown in MTCT following option-B+ implementation, MTCT remains high in Cameroon, with substantial disparities across regions. Thus, in this era of option-B+, achieving MTCT elimination requires interventions in northern-Cameroon. The variation in pro-viral load in infected-children underlines the relevance of characterizing viral reservoirs for possible infection control in tropical settings.

## Introduction

About 38 million people were living with Human Immunodeficiency Virus (HIV) worldwide, according to the 2019 UNAIDS report; sub-Saharan Africa (SSA) is still paying the heaviest toll, since nearly 70% of the world’s recorded HIV infections is found in this part of the globe [1]. Importantly, among the 150,000 children (0–14 years) who have been newly infected in 2019, 126,000 (84%) are living in SSA [1]. In Cameroon, an estimated 7,600 new cases pediatric HIV infection occur yearly, placing Cameroon among the priority countries for programmatic interventions against HIV vertical transmission [2].

Vertical transmission of HIV-1 also known as mother-to-child transmission (MTCT) of HIV is one of the HIV transmission route, and represents the major route through which pediatric populations acquire HIV-1 infection [3]. This can occur during pregnancy, birth or through breast feeding where nearly 35% of children born to HIV-1 positive mothers contract HIV infection [2]. More than 90% of HIV-1 infected children acquired the infection through MTCT [4, 5], especially in SSA [6]. Studies reported that, during pregnancy or postpartum period, the rate of MTCT varies from 15% to 45% in the absence of prevention of MTCT (PMTCT) intervention [2, 7]. However, with the expansion of PMTCT interventions, a significant reduction of MTCT rate from 28% to 18% was observed in SSA between 2009 and 2013 [8].

In Cameroon, analysis of the PMTCT cascade at the end of 2012 revealed some shortcomings, both in the demand for PMTCT services and in the quality of services offered in health facilities throughout the country [2]. In 2014, Cameroon PMTCT strategy was based on three main areas of interventions [2], which includes the integration of PMTCT program; the enhancement of maternal, neonatal and child health program; and the last consists of tasks shifting/decentralization of services and the implementation of option B+ for PMTCT, which recommends the systematic initiation of antiretroviral therapy (ART) in all HIV-1-positive pregnant women regardless of their clinical stage or CD4 cell-count [2].

Efforts to study the prevalence and risk factors of MTCT have been made, but primary studies in Cameroon remain highly diverse and challenging for guiding toward evidence-based decision [9–15]. Therefore, this systematic review and meta-analysis intended to review the existing evidence and to determine the pooled national estimate of MTCT rate in Cameroon, and its associated factors. Moreover, for HIV-infected children within the frame of PMTCT, our work also aimed at summarizing the existing data on HIV-DNA viral reservoirs profile following vertical transmission in Cameroon. The results of this study could contribute substantially to generate stronger recommendations for policy-making and public health actions on both the preventing MTCT while ensuring an effective infection control for those that might be infected at county-level.

## Methods

### Design

This systematic review and meta-analysis was performed following the guidelines of preferred Reporting Items for Systematic Review and Meta-Analyses (PRISMA) (S1 Table) [16] and was registered to PROSPERO under the registration number CRD42021224497.

### Data sources and search strategy

To enable exhaustive identification of relevant studies, a comprehensive search strategy was performed in the PubMed, African Journals Online, Google scholar, ScienceDirect and academic medical education databases using the search terms: “Human immunodeficiency virus”; HIV; “Acquired immunodeficiency syndrome”; AIDS; “mother to child”; “mother-to-child”; “mother to infant”; “vertical transmission”; “mother-to-child-transmission”; “mother to child transmission”; MTCT; PMTCT; “Prevention of mother to child transmission”; “Early infant diagnosis”; “viral reservoirs”, Centre; South; Littoral; West; “North West”; “South West”; East; Adamaoua; North; “Far North” and Cameroon. Search run was developed using the Boolean operators “AND” and “OR”. Search details for PubMed illustrated as example is shown in S2 Table. Databases were consulted from February to August 2021 for studies published in English or French languages. Furthermore, reference lists of included articles were searched manually to ensure the completeness of the search strategy.

### Inclusion and exclusion criteria

The included studies had the following criteria: (a) interventional or observational studies published in peer-review journals including epidemiological surveys, cross-sectional studies, cohorts, case-control and case series or reports (with sample size ≥10 participants); grey literatures (abstract for cconferences, government reports) reporting expected data were also considered as an inclusion criteria; (b) studies with mother-to-child HIV transmission as the main outcome; (c) studies with any HIV diagnosis approach among exposed infants including early infant diagnosis (EID) for infant aged <18 months and serological test as recommended nationally for infants aged 18 months and above; (d) Studies reporting the prevalence of HIV (serological and/or EID) among HIV exposed infants born to HIV positive mothers or studies with available data to calculate this estimate were included. We defined MTCT as the proportion of the number of infants positive for HIV divided by the total HIV-exposed infants assessed. Regarding cohort studies, the cumulative incidence was considered as prevalence, in which the number of new HIV infected cases was divided by the overall sample size. Studies focusing on the assessment of knowledge, attitude, and practice of MTCT without the outcome of interest of our study, case reports, reviews, systematic reviews and meta-analyses, studies with outliers’ data, comments, studies without full text, and duplicate were excluded.

### Study selection and quality assessment

The list of selected studies was exported to an excel spreadsheet. Duplicates identified from the complete list of studies were removed. The titles and abstracts of the eligible studies were independently examined by two study authors (ACK and ADN) for the selection of relevant studies. The contrary opinions of the investigators regarding the selection of the studies were resolved by discussion, consensus or intervention of a third person (experienced scientist) when necessary.

The quality of each study was independently assessed by three study authors (ACK, AND and ENJS) using a dedicated scale for prevalence studies that is based on 10 components divided into two groups: internal and external validity of the study. This scale was adapted by removing item 9 (S3 Table) [17]. The scores of 0 or 1 were assigned to each question in the assessment tool for a total score of 9 per study. The scores of 0–3, 4–6 and 7–9 represented a high, moderate and low risk of bias, respectively.

### Data extraction

Data from the included studies were extracted using a Google form by 03 study authors (ACK, AND and ENJS) and verified by ACK. The extracted data were: the name of the first author, the year of publication, the study design, the inclusion criteria, region, sampling method, sampling period, age, gender, sample size, the prevalence rate of MTCT, the feeding mode and the mode of delivery. Disagreements observed by different data extractors during data extraction were resolved by discussion and/or consensus. The corresponding authors of the selected studies were contacted for further information whenever pertinent data for the analysis were missing.

### Data analysis

Before performing meta-analysis, outliers were identified and removed (www.Statology.org/remove-outliers-r/). To estimate the heterogeneity among studies, I^2^ and H statistics were used [18]. The I^2^ value was indicative of the degree of heterogeneity, with values of 0%, 18%, 45%, and 75% designating none, low, moderate and high heterogeneity, respectively [19]. Lack of evidence on heterogeneity among studies was indicated by obtaining an H statistic close to 1, these values were inversely proportional with the degree of heterogeneity. The prevalence and 95% confidence intervals (95% CI) were estimated by random effect models [20]. Subgroup and meta-analyses according to the study design, geographical area, demographic situation, presence/absence of PMTCT intervention, mode of delivery and mode of feeding were employed to adjust for the variations in the pooled estimate of the prevalence. The dependent variable was the prevalence of HIV-1 MTCT. The statistically significant threshold was fixed at p<0.05. The publication bias was assessed by visual inspection of the asymmetry of the funnel plot and the Egger test, with p *<*0.1 indicating a potential bias [21]. The *R version 3*.*6*.*0* software (package "meta" and "metafor") was used to perform all meta-analyses, through the R Studio interface [22, 23].

## Results

### Literature search

**Fig 1:**
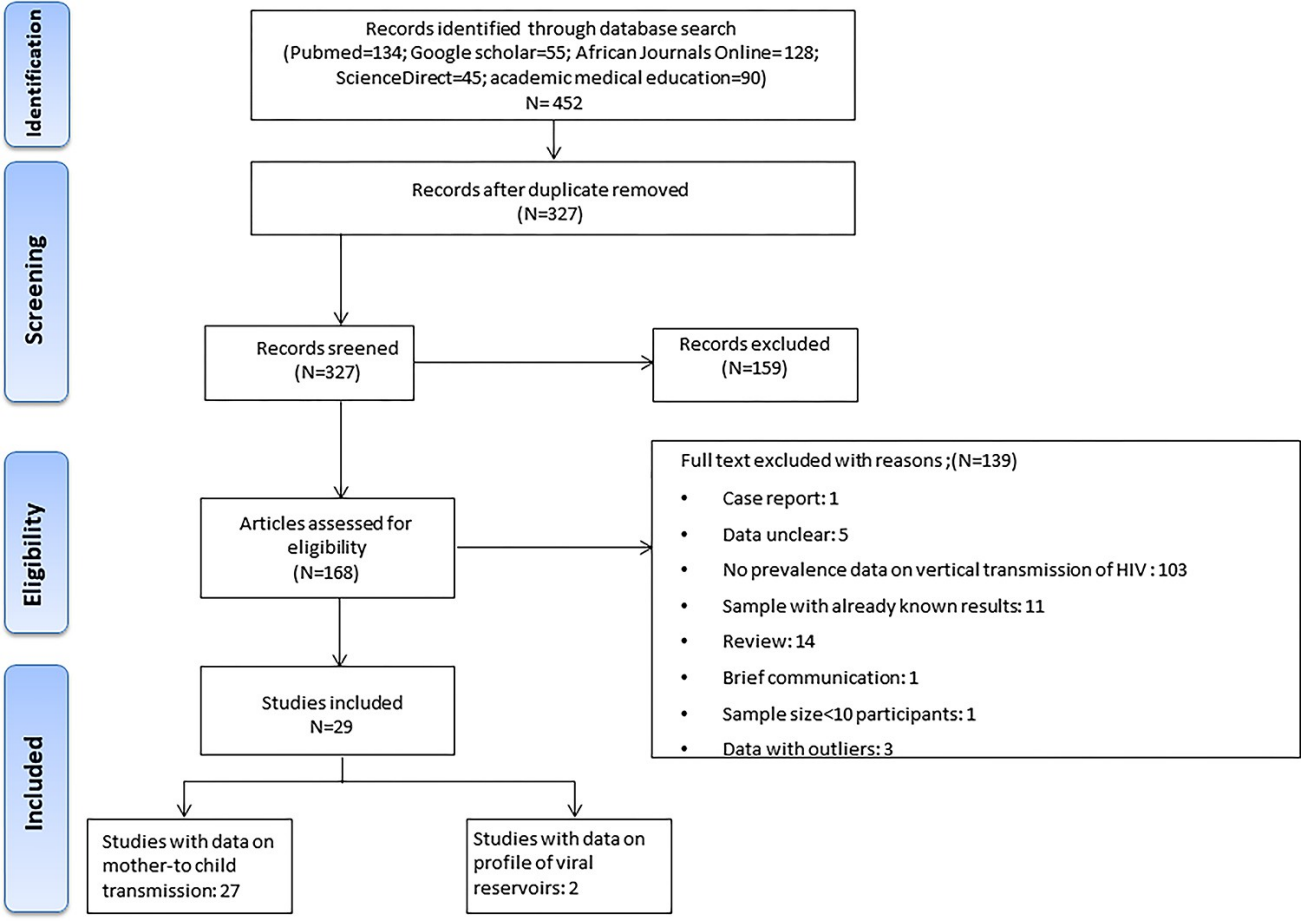
Flow chart of the study selection process.

A total of 452 studies were identified through an electronic search strategy in the different databases. First, duplicates (n = 1256), irrelevant studies based on titles and abstracts were removed (n = 159) and 168 studies were assessed eligible for the full text examination. After this process, 29 studies met all the inclusion criteria. Fig 1 shows the study selection process and S4 Table shows the main reasons for excluding initially eligible studies.

### Characteristics of included studies

Among 28 studies included, we found 11 cohort studies [13, 24–33] and 17 cross sectional studies [9–11, 14, 34–46]. Study participants were recruited between 1994 and 2017. Regarding geographical location, all the ten regions of Cameroon were represented; one in the Adamoua [30]; 19 in the Centre [13, 14, 24, 25, 27, 29, 30, 32–35, 40, 42–49]; two in the East [10, 30]; one in the Far North [30]; three in the Littoral [28, 30, 35]; one in the North [30]; four in the North-West [11, 30, 35, 38]; one in the South [30]; four in the South-West [28, 30, 31, 35]; and two in the West [26, 30]. Concerning the types of studied population, most of the studies were conducted in the urban area [9–11, 13, 14, 24, 25, 27, 29, 31–34, 37, 42–47, 49]; while only three of the studies were conducted in the rural area [26, 28, 38]. The characteristics of those studies are reported in Table 1.

**Table 1:** General characteristics of the included studies for prevalence of MTCT and quality of assessment.

Author, year	Years of sampling	Study Design	Sample size	MTCT Positivity rate (95%CI)	Infant median/mean age in week	Quality assessment
Tscherning *et al*., 2000 [44]	1994–1996	Cross sectional	42	11.90% (3.98–25.63)	Not Reported	Moderate risk of bias
Ayouba *et al*., 2003 [45]	2000–2002	Cross sectional	123	4.07% (1.33–9.23)	Not Reported	Low risk of bias
Tejiokem *et al*., 2004 [47]	2000–2004	Cross sectional	313	13.10% (9.57–17.35)	Not Reported	Low risk of bias
Wanyu *et al*., 2007 [38]	2002–2005	Cross sectional	14	14.29% (1.78–42.81)	Not Reported	Moderate risk of bias
Kouam *et al*., 2006 [42]	2003–2004	Cross sectional	18	11.11% (1.38–34.71)	Not Reported	Moderate risk of bias
Tchendjou *et al*., 2010 [33]	2004–2008	Cohort	418	7.18% (4.89–10.09)	Not Reported	Low risk of bias
Boerma *et al*., 2015 [28]	2004–2012	Cohort	285	3.86% (1.94–6.80)	Not Reported	Low risk of bias
Nkenfou *et al*., 2019 [9]	2004–2013	Cross sectional	15404	9.35% (8.89–9.82)	16.7	Low risk of bias
Fomulu *et al*., 2009 [49]	2006–2006	Cross sectional	90	1.11% (0.03–6.04)	Not Reported	Low risk of bias
Lukong *et al*., 2013 [31]	2007–2008	Cohort	174	6.90% (3.61–11.74)	6	Low risk of bias
Tejiokem *et al*., 2011 [29]	2007–2009	Cohort	1331	3.83% (2.87–5.01)	0.4	Low risk of bias
Penda *et al*., 2019 [24]	2007–2010	Cohort	1765	3.85% (1.22–5.46)	0.42	Low risk of bias
Nkenfou *et al*., 2012 [30]	2007–2010	Cohort	14763	9.84% (9.36–10.33)	Not Reported	Low risk of bias
Tejiokem *et al*., 2015 [27]	2007–2011	Cohort	1971	10.65% (9.33–12.10)	Not Reported	Low risk of bias
Njom *et al*., 2013 [25]	2008–2009	Cohort	285	2.81% (1.43–5.67)	8.0	Low risk of bias
Njom *et al*., 2012 [32]	2008–2010	Cohort	14763	4.55% (2.87–6.80)	Not Reported	Low risk of bias
Njom *et al*., 2018 [13]	2008–2013	Cohort	1086	3.59% (2.57–4.88)	Not Reported	Low risk of bias
Fondoh *et al*., 2017 [11]	2008–2014	Cross sectional	877	7.07% (5.46–8.97)	6.0	Low risk of bias
Altan *et al*., 2016 [26]	2009–2011	Cohort	265	1.13% (0.23–3.27)	Not Reported	Low risk of bias
Noubiap *et al*., 2013 [10]	2010	Cross sectional	112	11.61% (6.33–19.03)	16.0	Low risk of bias
Temgoua *et al*., 2015 [36]	2010–2011	Cross sectional	3789	11.45% (10.46–12.51)	14.0	Low risk of bias
**Pooled prevalence before Option B+**	**-**	**-**	**57888**	**8.97% (8.71–9.24)**	-	-
Njom *et al*., 2019 [14]	2016	Cross sectional	120	5.83% (2.38–11.65)	60.0	Low risk of bias
Tchendjou *et al*., 2020 [35]	2016–2017	Cross sectional	2254	8.74% (7.61–9.98)	7.0	Low risk of bias
**Pooled prevalence after Option B+**	-	-	**2374**	**2.88% (5.03–9.34)**	-	-
Mekue *et al*., 2018 [34]	Not Reported	Cross sectional	220	19.09% (14.12–24.92)	Not Reported	Low risk of bias
Ayouba *et al*., 2003 [40]	Not Reported	Cross sectional	119	10.92% (5.95–17.96)	Not Reported	Low risk of bias
Ou *et al*., 2007 [41]	Not Reported	Cross sectional	315	15.56% (11.74–20.04)	9.4	Low risk of bias
Njom *et al*., 2010 [43]	Not Reported	Cross sectional	47	4.26% (0.52–14.54)	Not Reported	Moderate risk of bias

**Notes**: The risk of bias assessment was evaluated following the guidelines reported by Hoy et al., 2012 [17]

The Prevalence was calculated using the data reported in the different articles.

Option B+ was implemented as from 2014.

### Meta-analysis

The included studies of this review recruited a total of 46 684 infant participants aged between 0.4 and 60 weeks regarding MTCT and 136 children with data reported on profile of viral reservoirs.

#### Publication bias and heterogeneity analysis

**Fig 2:**
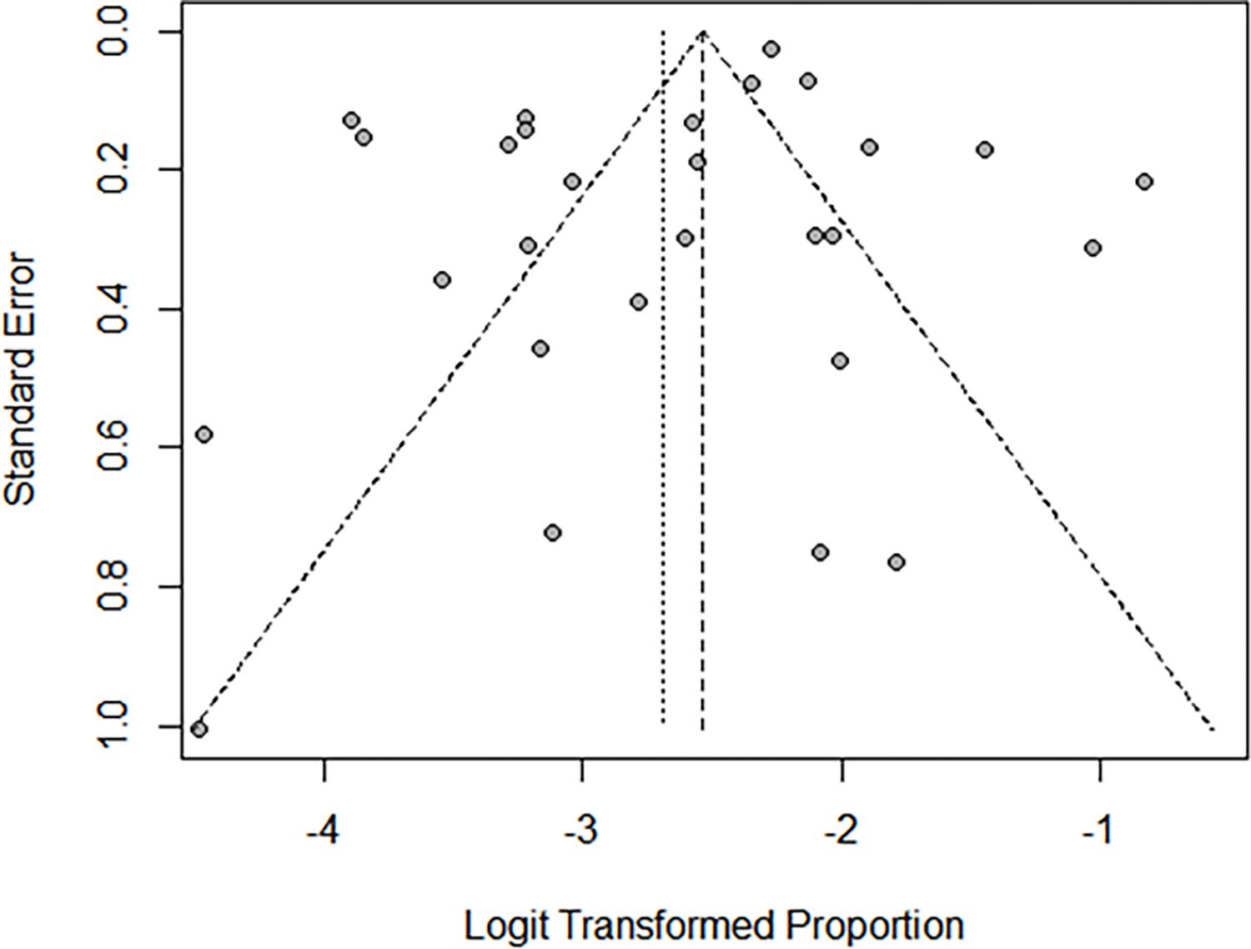
Funnel plot for publication bias.

No study was excluded after checking for the funnel plot symmetry and the significance of Egger’s regression test (p = 0.103, which indicated an absence of publication bias) (Fig 2). PMTCT subgroup category among both children and their mothers had a small number of studies and did not enable for an objective estimate of the heterogeneity. Nonetheless, substantial heterogeneity was recorded in both cross sectional, cohort studies and other subgroup analysis.

#### Prevalence of mother to child transmission

**Fig 3:**
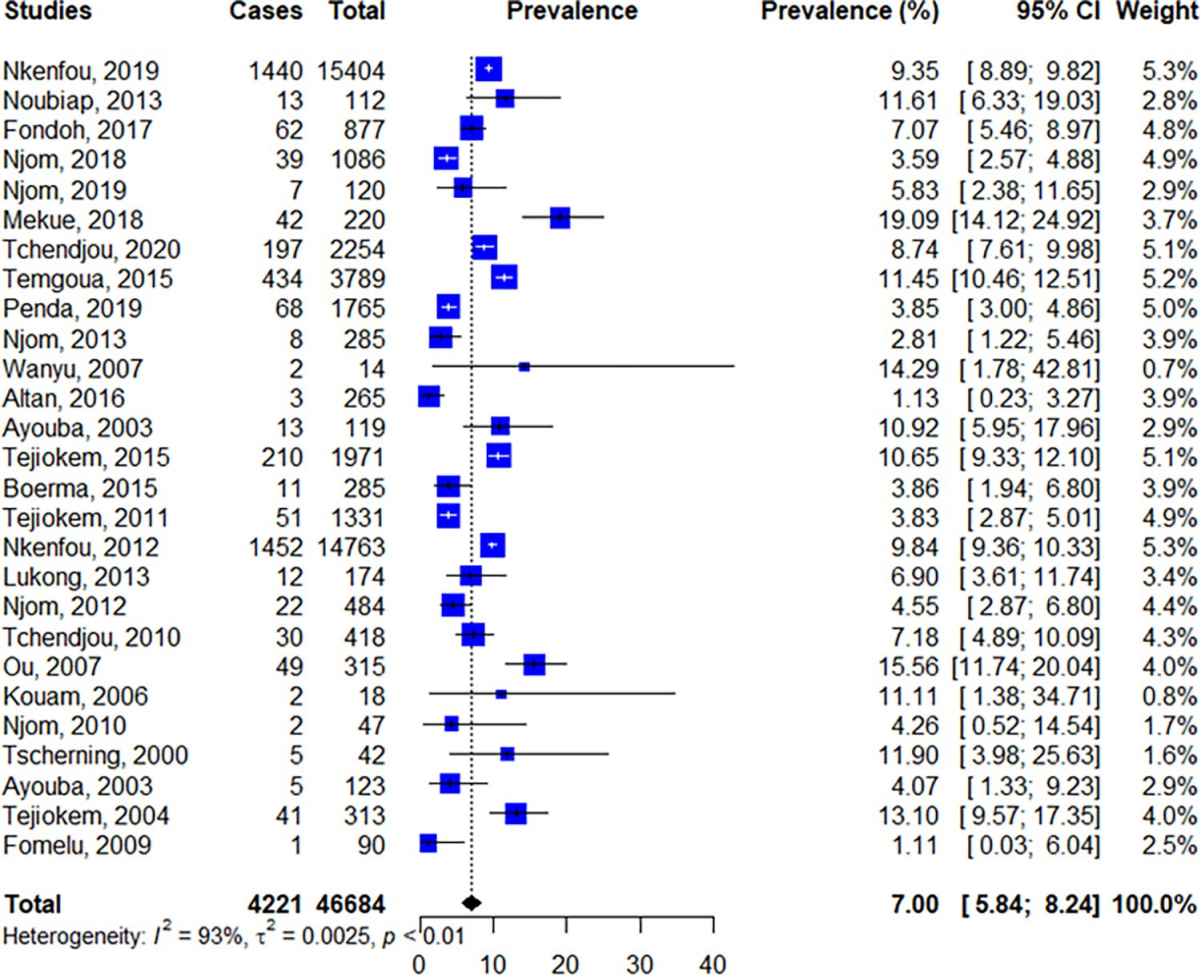
Forest plot of the prevalence of MTCT.

Out of the 29 included studies, 27 studies reported data on MTCT and were considered in the final meta-analysis [9–11, 13, 14, 24–46]. The pooled prevalence (95% confidence interval) rate of MTCT was 7.00% (6.07–8.51), I^2^ = 93%, p<0.01 (Fig 3).

**Fig 4:**
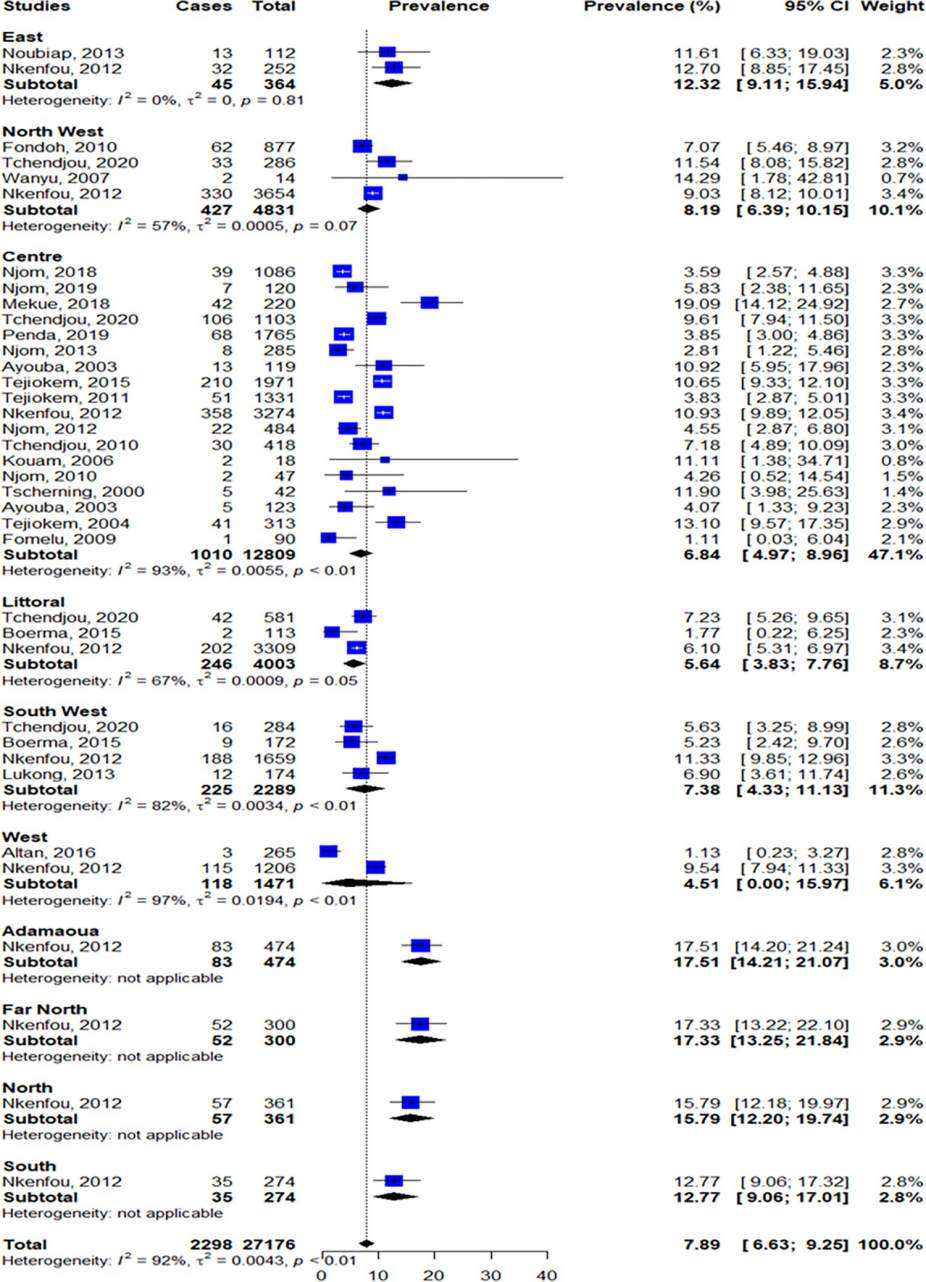
Forest plot of MTCT in the ten regions of Cameroon. The number of participants obtained in this forest plot depend on the studies that reported the region of sampling.

**Fig 5:**
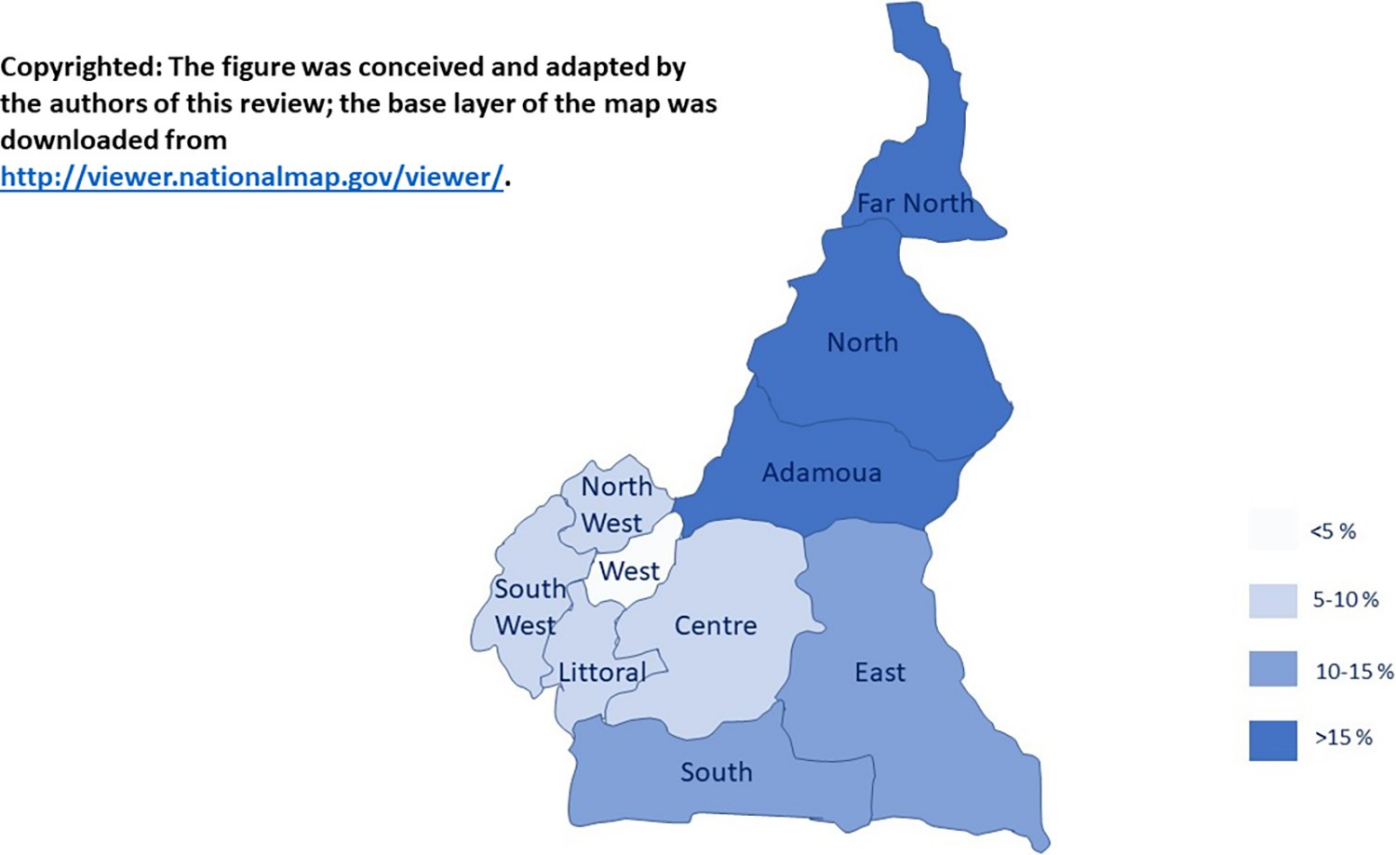
Pooled vertical transmission rate of HIV in Cameroon. The figure was conceived and adapted by the authors of this review; the base layer of the map was downloaded from http://viewer.nationalmap.gov/viewer/.

Moreover, we have performed a subgroup analysis by region. Globally, the Northern part of the country showed the highest MTCT prevalence [95% CI]; with a prevalence of 17.51% (14.21–2 1.07) in the Adamaoua region; 17.33% [13.25–21.84] in the Far North region and 15.79% [12.20–19.74] in the North region). The South and the East regions were also highly affected with a prevalence (95% CI) of 12.77% (9.06–17.01) and 12.32% (9.11–15.94) respectively. The West region was the least affected, with a prevalence of 4.51% (0.00–15.94) of prevalence; and thus is the only one heading toward MTCT elimination. Furthers information are reported in Figs 4 and 5.

**Fig 6:**
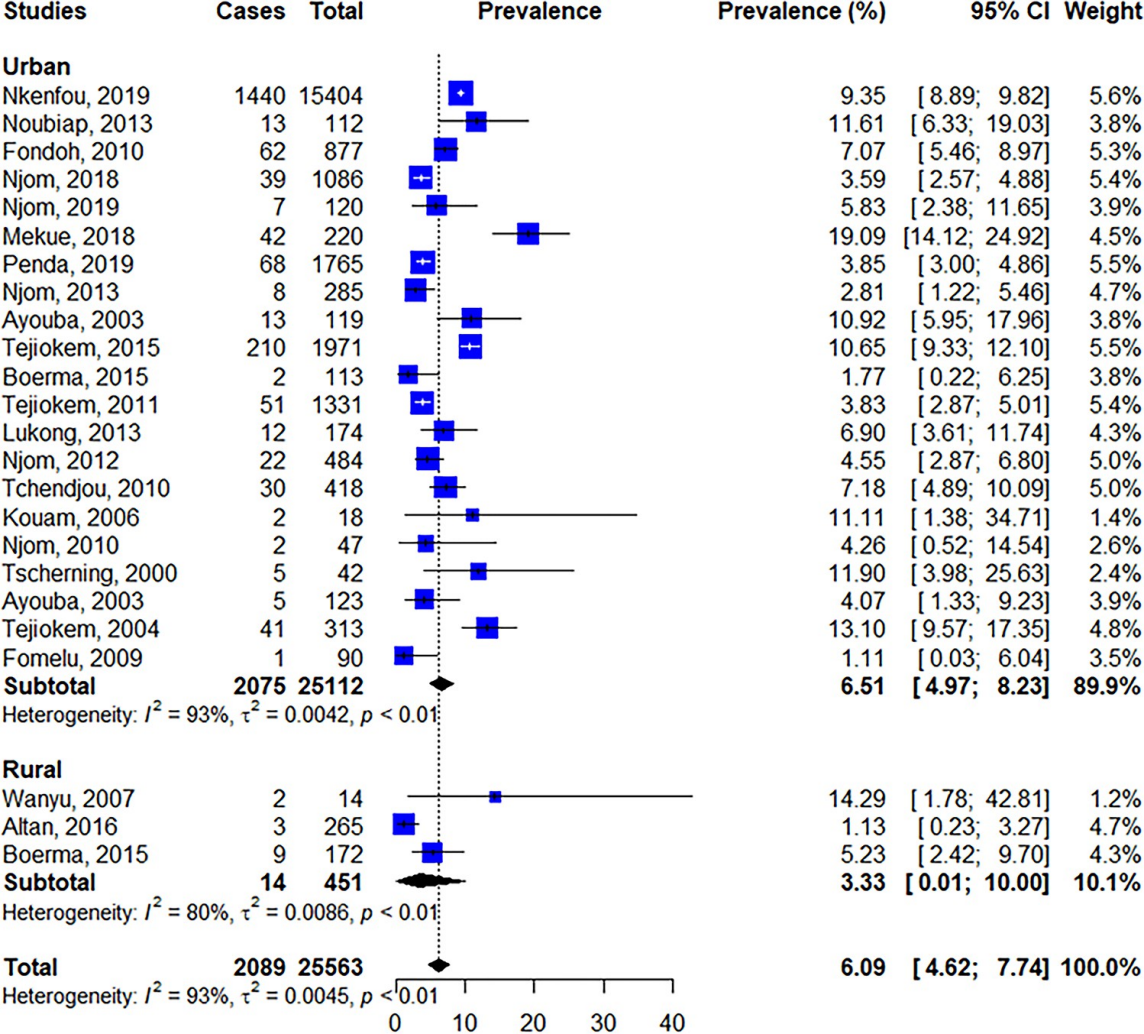
Forest plot of the prevalence of MTCT according to the demographic area.

Regarding demographic situation, the Urban zone with 21 studies showed a higher prevalence of MTCT (6.92% [95% CI:5.33–8.69]; I^2^ = 93%, P<0.01), when compared to rural area with 03 studies (3.33% [95% CI: 0.01–10.00], I^2^ = 80%, P<0.01) (Fig 6). Of note, three studies did not report demographic information.

**Fig 7:**
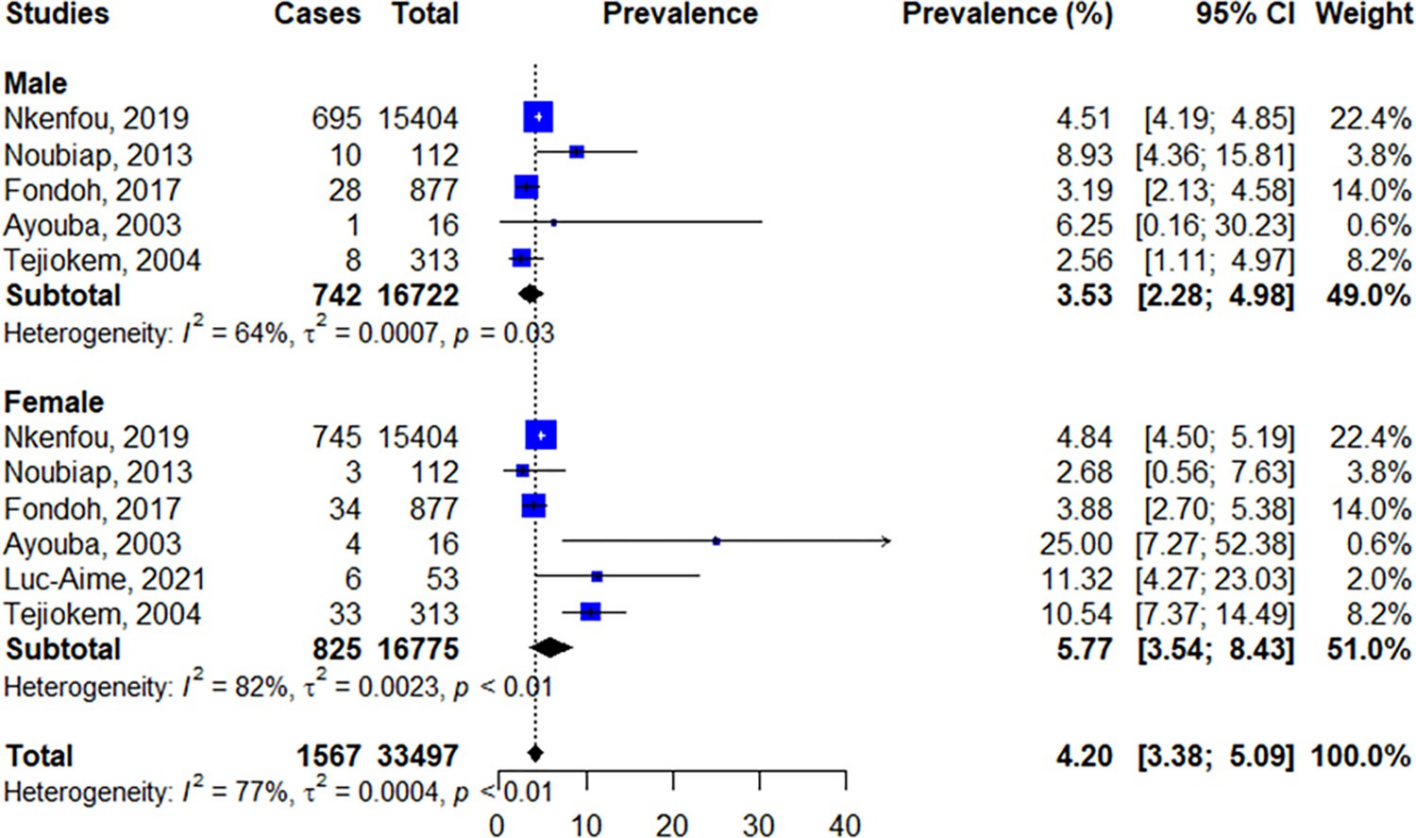
Forest plot of the prevalence of the MTCT according to gender.

Regarding gender, female children showed a higher prevalence rate (5.77% [95% CI: 3.54–8.43]; I^2^ = 82%, p<0.01) when compared to male children (3.53% [95% CI: 2.28–4.98]; I^2^ = 64%, p = 0.03); Fig 7.

**Fig 8:**
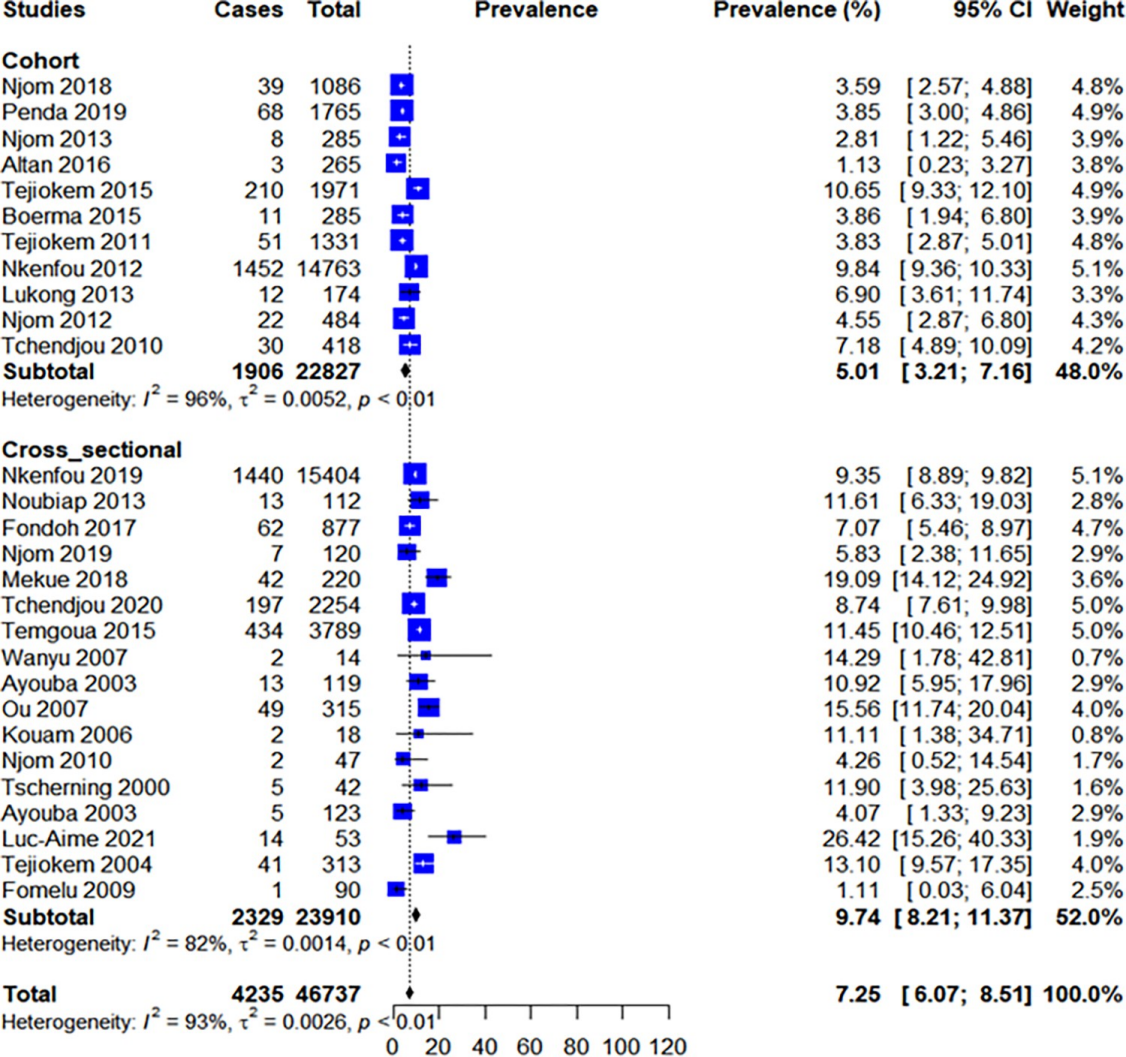
Forest plot of the prevalence of the MTCT according to study design.

A subgroup analysis was also performed according to study design. We found that a higher prevalence of 9.74% (95% CI: 8.21–11.37) was observed in cross sectional studies (p<0.01, I^2^ = 82%), versus 5.01% (95% CI: 3.21–7.16) in cohort studies (p<0.01, I^2^ = 93%); Fig 8.

#### Factors associated with mother to child transmission

The main risk factors considered in this review were: (a) Presence of the prevention of MTCT (PMTCT), (b) feeding modes and (c) delivery route.

**Fig 9:**
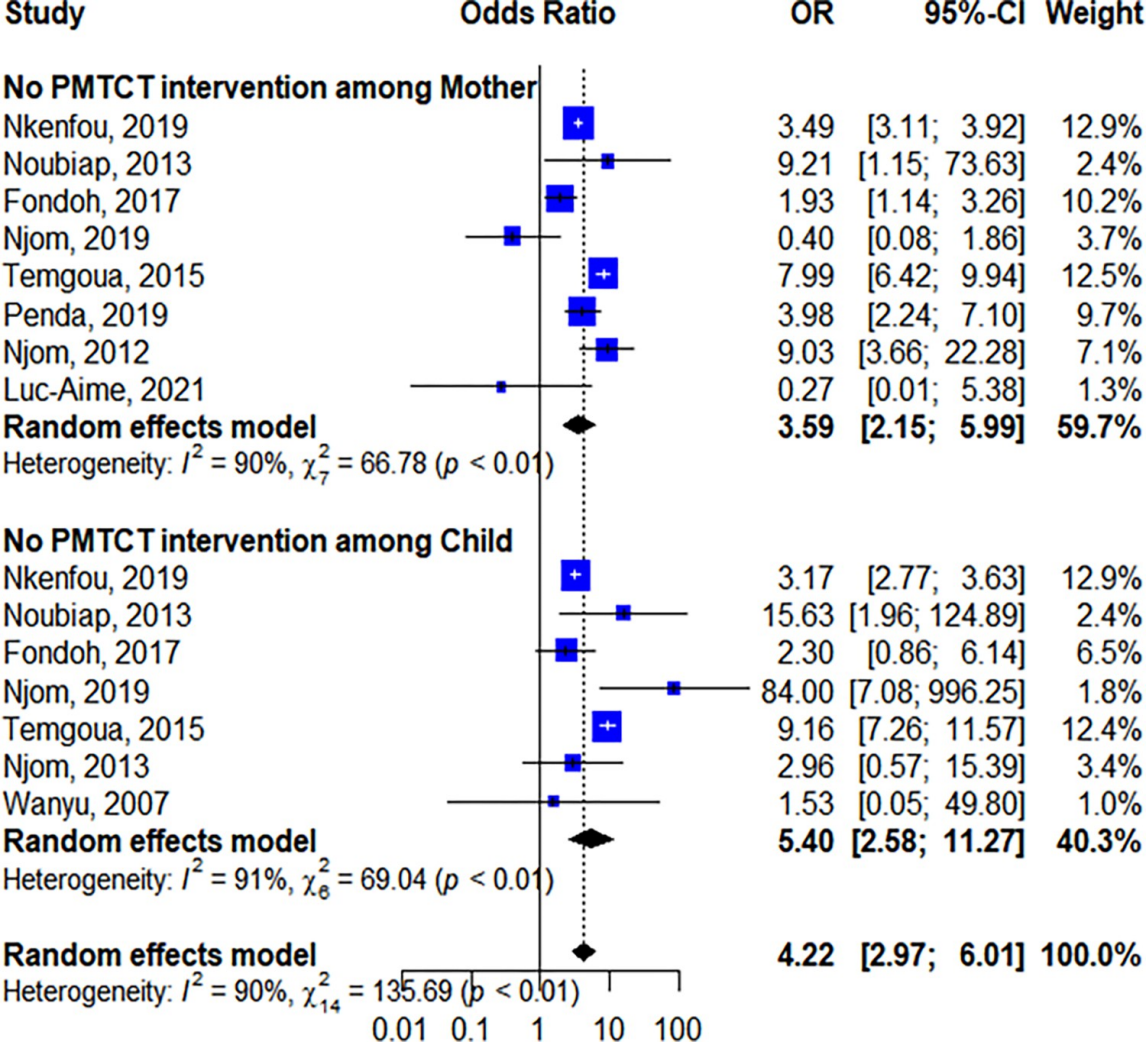
Association between MTCT of HIV and prevention of mother-to-child transmission intervention.

MTCT was associated with the absence of PMTCT intervention among children (OR: 5.40 [95% CI: 2.58–11.27]) and mothers (OR:3.59 [95% CI: 2.15–5.99]) as shown in Fig 9. In this study, there was no association between MTCT and other feeding modes (OR:1.55 [95% CI: 0.92–2.64]) and delivery route (OR: 0.95 [95% CI: 0.60–1.51] for vaginal delivery mode and 0.86 [95% CI: 0.45–1.63] for cesarean delivery mode]) (S1 Fig).

#### Evaluation of the PMTCT-option B+ strategy

Regarding Cameroon’s strategy to prevent MTCT, PMTCT-option B+ resulted in almost 25% reduction in MTCT, dropping from 8.97% (95% CI: 8.71–9.24) in the absence of option B+ to 2.88% (95% CI: 5.03–9.34) with option B+; see Table 1 for further information.

#### Profile of viral reservoirs among infected children

Out of the 29 studies included in this review, only two reported data on the profile of cellular reservoirs among 136 enrolled children. A pro-viral DNA load mean of 3.34±1.05 log_10_/mL without genotypic characterization was observed among five HIV infected children over 57 enrolled during the ANRS-Pediacam study in 2007 [50]. As for the genotyping aspect in the Cameroonian context, archived HIV drug resistance mutations were identified exclusively in pro-viral DNA marker among 21 of 79 adolescents vertically HIV-infected failing antiretroviral therapy and enrolled in the Centre health facilities in 2019 [51]. Very limited studies have been conducted on the viral reservoirs of HIV-infected children in Cameroon, thus calling for further investigations in this area of scope.

## Discussion

This systematic review and meta-analysis aimed to determine the pooled prevalence of MTCT, its determinants and the profile of viral reservoir among HIV-1 infected children in Cameroon. Several lessons and prospects could be learnt from the trend of evidence.

The overall positivity rate of MTCT in Cameroon was 7.00% and varied across the regions. The positivity rate of MTCT in the present study was higher than the 6% and 2% respectively observed in South Africa and Botswana as reported by 2013 United Nations Program on HIV/AIDS [52] reflecting a potential low maternal adherence to antenatal care utilization in Cameroon. Low level of knowledge and practices of mothers about MTCT might contribute to the high HIV infection rate among infants in our setting [53]. By contrast, this rate was lower when compared to those reported among HIV exposed in some African countries such as Ethiopia (11.4%) [54]. This discrepancy might be due to the sampling year or may perhaps reflect the differences in PMTCT uptake between the settings.

Regarding sub-group analysis of MTCT rate according to geographical areas, the Northern part of the country (Adamaoua, Far North and North regions) showed a higher transmission rate, followed by South and East regions. Among the ten regions, the West showed the lowest MTCT transmission rate. These differences could be explained by the sub-optimal and late start of antenatal visits. Therefore, most children born to HIV-positive mothers, particularly in the northern part of the country, because they are not diagnosed (or not early diagnosed), would be without treatment and therefore potentially reach the advanced WHO clinical stages (III or IV) because of their high viral replication and immunodeficiency. Indeed, this study shows that in the northern part of the country, there could be a high risk of HIV-related infant morbidity/mortality. In fact, it was previously documented that the Northern regions of Cameroon have critical shortage in human resource for health and that more than 60% of births are unattended [55]. Moreover, these regions record the highest number of childbirths in Cameroon [56]. For these reasons, women have limited access to HIV testing [53]. The lower awareness/knowledge levels of pregnant women about HIV, HIV transmission and PMTCT are important determinants of MTCT [15]. So as potential strategies, it is necessary to raise awareness and promote the use of PMTCT, increase healthcare workers, broaden access to demedicalised HIV screening with “same-day test and result” strategy and increase the knowledge of pregnant women about all aspects of HIV. Moreover, because HIV-infected pregnant women are undergoing psychological distress, healthcare must provide psychosocial support and accompaniment using a friendly approach. For rural area especially in the northern part of the country, an increasing antenatal care coverage with free access might improve access to PMTCT. Of note, this huge difference between the Northern and the Southern part of the country was especially observed before the implementation of option B+, demonstrating the need of updating the MTCT prevalence data in this part of the country.

Concerning the settings (rural and urban), our study showed a high positivity rate of MTCT in urban zone when compared to rural area (Fig 6). This observation was unexpected because in general, a poor patients’ monitoring, recurrent ARV stock out and poor ART adherence is common in rural settings [57]. The higher rate in urban settings here should be interpreted with caution as most of the included studies in this review were conducted in urban settings (22 studies) versus only three studies in the rural settings. This result demonstrates the need of conduct more surveys related to MTCT in order to accurately estimate the burden of MTCT in rural areas and identify its associated risk factors.

Regarding gender, female infants had a higher positivity rate when compared to male infants, this is mostly attributed to the increased risk of intrauterine HIV infection in female children [58].

Despite the 25% reduction observed in MTCT following the implementation of Option B+ as from 2014 in Cameroon, this review shows that, the main predictor of MTCT remains the absence of PMTCT interventions. HIV exposed infants without exposure to any form of PMTCT intervention for prophylaxis had 5.40 odds to acquire HIV infection. This finding was in line with a study conducted in similar settings [59], suggesting that not initiating infant ART prophylaxis is indeed an important risk factor for MTCT [60, 61]. Similarly, infants born from mothers who did not receive PMTCT had 3.59 odds of being infected by HIV. This finding is also in line with studies conducted in Ethiopia and Kenya where infants whose mother couldn’t get PMTCT interventions had 5.10 odds of HIV transmission [54, 62, 63]. This might be due to the fact that without maternal ART during pregnancy, active viral replication could favor MTCT [59, 63], and longer duration of ART during pregnancy was associated with suppressed viral load at delivery [64].

Our data did not show an association between MTCT and mixed feeding mode, but many studies previously reported an association between MTCT and mixed feeding mode. Our findings were unexpected because it is already known that mixed feeding mode may cause laceration of gastrointestinal mucosa which promotes a favorable environment for the viral entry into the bloodstream and then to the target cells [65]. This lack of association in our analysis could be mainly due to insufficient and/or erratic data. In the same vein, no significant association found between MTCT and delivery route and again, this could be explained by the insufficient data. Recent studies are therefore needed to provide more data on this aspect, especially in the context of the highly potent and modern ART. Cameroon is one of the priority countries for the elimination of MTCT, but to reach this goal, it will be important to improve on other key determinants of health such as education (of especially women and young girls), healthcare access, economic empowerment of the communities and other potential modifiable factors [66, 67]. For example, improvement in the sensitization and case detection through technological innovation could play a crucial role in addressing some of the unmet medical needs related to PMTCT [66, 68].

Lastly, data on the profile of HIV-1 reservoirs especially regarding genotypic part in pediatric populations represent a major gap in the Cameroonian context. This observation shows that more research is warranted to characterize the HIV-1 reservoir among pediatric population, which is still of concern in developing countries. This is a disturbing concept in settings like Cameroon and SSA where there is an increasing number of children living with HIV, and ensuring a safe lifelong treatment for this vulnerable population requires the development of strategies to ensure a long-term control of viral replication or a functional cure. Implementing such investigation warrants the identification of research priorities on the genotypic patterns of cellular reservoirs, functional properties of latent versus active viral sanctuaries, and attempts toward the implementation of clinical trials for a long-term control of HIV infection in the population of vertically-infected children in SSA.

Our study has some limitations. As it is the case with most systematic review and meta-analysis design, the lack of some data and the heterogeneity across studies might have confounded some of our results. The time-trend analysis was not performed because of the lack of prevalence data in some years. Also, our results might not be fully representative due to the scarcity of studies in some regions.

## Conclusion

The pooled prevalence of MTCT in Cameroon is high (about 7%), with considerable geographical disparities across regions. Poor PMTCT coverage and sub-optimal interventions both in the child and the mother are associated with an increased MTCT rate. Despite its decrease following the implementation of Option B+, Cameroon still has a long way in eliminating MTCT. Specific interventions for ensuring elimination of MTCT should focus on the northern regions of the country, and sex-vulnerability in vertical transmission. Furthermore, remain a major gap is the lack of community-provision of ART to infected mothers and their children. For infected children, effective and lifelong viral control in a SSA country like Cameroon required a further understanding of the diversity of viral reservoirs and strategies toward a functional cure.

## Supporting information

S1 Fig Association between MTCT of HIV and feeding mode/childbirth delivery mode. (TIF)

S1 Table Preferred reporting items for systematic reviews and meta-analyses checklist. (DOCX)

S2 Table Search strategy in Medline (Pubmed). (DOCX)

S3 Table Items for risk of bias assessment. (DOCX)

S4 Table Main reasons of exclusion of eligible studies. (DOCX)

## List of abbreviation

AIDS: Acquired Immunodeficiency Syndrome
ART: Antiretroviral treatment
ARV: Antiretroviral
CI: Confidence Interval
HIV: Human Immunodeficiency Virus
MTCT: Mother-to-Child Transmission
OR: Odds Ratio
PMTCT: Prevention of Mother to Child Transmission
SSA: sub-Saharan Africa
