# Supplementary figures and images for: The mother-to-child transmission of HIV-1 and profile of viral reservoirs in pediatric population: A systematic review with meta-analysis of the Cameroonian studies
Source: PLoS One. 2023 Jan 17;18(1):e0278670. doi: 10.1371/journal.pone.0278670 (PMC9844886; doi:10.1371/journal.pone.0278670)

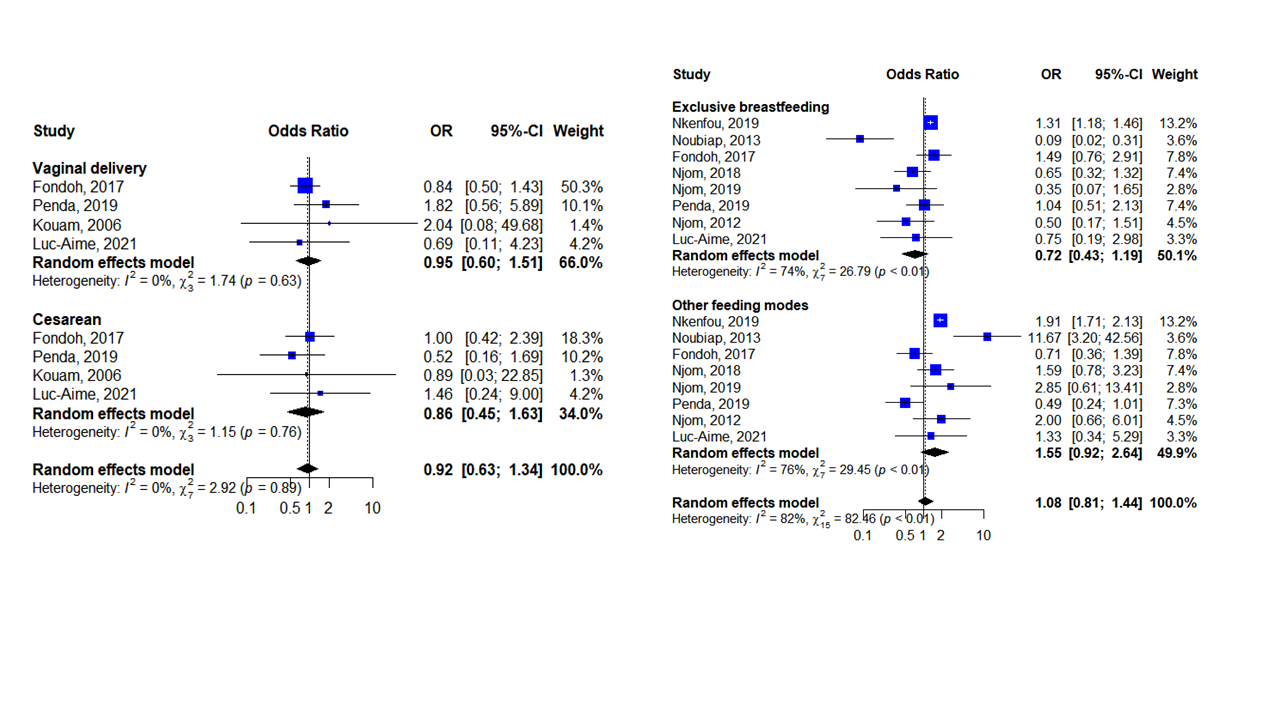

Supplement: S1 Fig — (TIF) [file pone.0278670.s001.tif]
