# Supplementary material for: The mother-to-child transmission of HIV-1 and profile of viral reservoirs in pediatric population: A systematic review with meta-analysis of the Cameroonian studies
Source: PLoS One. 2023 Jan 17;18(1):e0278670. doi: 10.1371/journal.pone.0278670 (PMC9844886; doi:10.1371/journal.pone.0278670)
Supplement: S2 Table — (DOCX) [file pone.0278670.s003.docx]

S2 Table: Search strategy in Medline (Pubmed)

| **Search** | **Virus** |
| --- | --- |
| #1 | “Human immunodeficiency virus” OR HIV OR “acquired immunodeficiency syndrome” OR AIDS; |
| #2 | “Mother to child” OR “mother to infant” OR “vertical transmission” OR “mother-to-child-transmission” OR MTCT OR PMTCT OR “Prevention of mother to child transmission” OR “Early infant diagnosis” OR “Viral Reservoirs” |
| #3 | Centre OR South OR Littoral OR West OR “North West” OR “South West” OR East OR Adamaoua OR North OR “Far North” OR Cameroon |
| #4 | #1 AND #2 AND #3 |
