# Supplementary material for: The mother-to-child transmission of HIV-1 and profile of viral reservoirs in pediatric population: A systematic review with meta-analysis of the Cameroonian studies
Source: PLoS One. 2023 Jan 17;18(1):e0278670. doi: 10.1371/journal.pone.0278670 (PMC9844886; doi:10.1371/journal.pone.0278670)
Supplement: S3 Table — (DOCX) [file pone.0278670.s004.docx]

S3 Table: Items for risk of bias assessment

| **Hoy *et al*. tool** | **Yes (1)/No (0)** |
| --- | --- |
| **External validity** |  |
| 1. Was the study’s target population a close representation of the national population in relation to HIV prevalence? | **1** |
| 2. Was the sampling frame a true or close representation of the study population? | **1** |
| 3. Was some form of random selection used to select the sample, OR was a census undertaken? | **1** |
| 4. Was the likelihood of nonresponse bias minimal (> 70%)? | **1** |
| **Internal validity** | **1** |
| 5. Were data collected directly from the subjects (as opposed to a proxy)? | **1** |
| 6. Was an acceptable case definition used in the study? | **1** |
| 7. Was the study viral detection assay shown to have validity and reliability? | **1** |
| 8. Was the same mode type of sample collected for all subjects? | **1** |
| 9. Was the length of the shortest prevalence period for the parameter of interest appropriate? | **1** |
| 10. Were the numerator(s) and denominator(s) for the HIV prevalence appropriate? | **1** |
| Total score | **10** |
| **Interpretation of the risk of bias tool** • 7-9: Low risk of bias • 4-6: Moderate risk of bias • 0-3: High risk of bias |  |

Hoy D, Brooks P, Woolf A, Blyth F, March L, Bain C, et al. Assessing risk of bias in prevalence studies: modification of an existing tool and evidence of interrater agreement. J Clin Epidemiol. 2012; 65: 934–939. https://doi.org/10.1016/j.jclinepi.2011.11.014 PMID: 22742910

Note: The Hoy D et al tool was adapted by removing item 9. Therefore, the total score was considered was 9 instead of 10.
