# Supplementary material for: The mother-to-child transmission of HIV-1 and profile of viral reservoirs in pediatric population: A systematic review with meta-analysis of the Cameroonian studies
Source: PLoS One. 2023 Jan 17;18(1):e0278670. doi: 10.1371/journal.pone.0278670 (PMC9844886; doi:10.1371/journal.pone.0278670)
Supplement: S4 Table — (DOCX) [file pone.0278670.s005.docx]

**S4 Table**: Main reasons of exclusion of eligible studies

| N° | Author, year of publication | Title | Reason of exclusion |
| --- | --- | --- | --- |
|  | Rao, 2019 | Predictors of early childhood HIV testing among children of sex workers living with HIV in Cameroon | Data unclear |
|  | Sama, 2017 | Prevalence of maternal HIV infection and knowledge on mother–to–child transmission of HIV and its prevention among antenatal care attendees in a rural area in northwest Cameroon | No data prevalence on vertical transmission of HIV in Cameroon |
|  | Yah, 2018 | Why is mother to child transmission (MTCT) of HIV a continual threat to new-borns in sub-Saharan Africa (SSA) | Review |
|  | Linguissi, 2019 | Prevention of mother-to-child transmission (PMTCT) of HIV: a review of the achievements and challenges in Burkina-Faso | Review |
|  | Penda, 2019 | Practices of Care to HIV-Infected Children: Current Situation in Cameroon | No data prevalence on vertical transmission of HIV in Cameroon |
|  | Budhwani, 2019 | HIV Status and Contraceptive Utilization among Women in Cameroon | No data prevalence on vertical transmission of HIV in Cameroon |
|  | Ngoufack, 2019 | CCR2 polymorphism and HIV: mutation in both mother and child is associated with higher transmission | Samples with already known results |
|  | Ford, 2018 | Maternal Decision-Making and Uptake of Health Services for the Prevention of Mother-to-Child HIV Transmission: A Secondary Analysis | No data prevalence on vertical transmission of HIV in Cameroon |
|  | Ikomey, 2017 | Observed HIV drug resistance associated mutations amongst naïve immunocompetent children in Yaoundé, Cameroon | Samples with already known results |
|  | Landefeld, 2017 | Prevention of Mother-to-Child Transmission of HIV in Yaounde: Barrier to Care | No data prevalence on vertical transmission of HIV in Cameroon |
|  | Biccard, 2018 | Perioperative patient outcomes in the African Surgical Outcomes Study: a 7-day prospective observational cohort study | No data prevalence on vertical transmission of HIV in Cameroon |
|  | Kufe, 2019 | Retention of health care workers at health facility, trends in the retention of knowledge and correlates at 3rd year following training of health care workers on the prevention of mother-to-child transmission (PMTCT) of HIV-National Assessment | No data prevalence on vertical transmission of HIV in Cameroon |
|  | Awungafac, 2015 | Prevention of mother-to-child transmission of the Human Immunodeficiency Virus: investigating the uptake and utilization of maternal and child health services in Tiko health district, Cameroon | No data prevalence on vertical transmission of HIV in Cameroon |
|  | Mouafo, 2017 | Host Molecular Factors and Viral Genotypes in the Mother-to-Child HIV-1 Transmission in Sub-Saharan Africa | Review |
|  | Chersich, 2018 | UNICEF's contribution to the adoption and implementation of option B+ for preventing mother-to-child transmission of HIV: a policy analysis | No data prevalence on vertical transmission of HIV in Cameroon |
|  | Bianchi, 2020 | Acceptability of Routine Point-of-Care Early Infant Diagnosis in Eight African Countries: Findings From a Qualitative Assessment of Clinical and Laboratory Personnel | No data prevalence on vertical transmission of HIV in Cameroon |
|  | Mekue, 2019 | HLA A*32 is associated to HIV acquisition while B*44 and B*53 are associated with protection against HIV acquisition in perinatally exposed infants | Samples with already known results |
|  | Bishop, 2019 | Maternal and neonatal outcomes after caesarean delivery in the African Surgical Outcomes Study: a 7-day prospective observational cohort study | No data prevalence on vertical transmission of HIV in Cameroon |
|  | Dambaya, 2019 | Differential expression of Fas receptors (CD95) and Fas ligands (CD95L) in HIV infected and exposed uninfected children in Cameroon versus unexposed children | Samples with already known results |
|  | Liotta, 2015 | Elimination of Mother-To-Child Transmission of HIV Infection: The Drug Resource Enhancement against AIDS and Malnutrition Model | Review |
|  | Luo, 2017 | Translating Technical Support Into Country Action: The Role of the Interagency Task Team on the Prevention and Treatment of HIV Infection in Pregnant Women, Mothers, and Children in the Global Plan Era | Review |
|  | Mavedzenge, 2017 | HIV infection among children and adolescents in Burundi, Cameroon, and the Democratic Republic of Congo | Samples with already known results |
|  | Tantchou, 2019 | The mother's carnet de santé (health booklet) in Cameroon: a tool for preventing mother-to-child transmission of HIV?() | No data prevalence on vertical transmission of HIV in Cameroon |
|  | Nkwabong, 2018 | Knowledge, attitudes and practices of health personnel of maternities in the prevention of mother-to-child transmission of HIV in a sub-Saharan African region with high transmission rate: some solutions proposed | No data prevalence on vertical transmission of HIV in Cameroon |
|  | Labhardt, 2009 | Early assessment of the implementation of a national programme for the prevention of mother-to-child transmission of HIV in Cameroon and the effects of staff training: a survey in 70 rural health care facilities | No data prevalence on vertical transmission of HIV in Cameroon |
|  | Luma, 2018 | Late presentation to HIV/AIDS care at the Douala general hospital, Cameroon: its associated factors, and consequences | No data prevalence on vertical transmission of HIV in Cameroon |
|  | Kalua, 2017 | Lessons Learned From Option B+ in the Evolution Toward "Test and Start" From Malawi, Cameroon, and the United Republic of Tanzania | Review |
|  | Bianchi, 2019 | Evaluation of a routine point-of-care intervention for early infant diagnosis of HIV: an observational study in eight African countries | Data unclear |
|  | Bissek, 2011 | Knowledge of Pregnant Women on Mother-to-Child Transmission of HIV in Yaoundé | No data prevalence on vertical transmission of HIV in Cameroon |
|  | Torimiro, 2018 | Rates of HBV, HCV, HDV and HIV type 1 among pregnant women and HIV type 1 drug resistance-associated mutations in breastfeeding women on antiretroviral therapy | No data prevalence on vertical transmission of HIV in Cameroon |
|  | Inzaule, 2020 | High levels of resistance to nucleoside/nucleotide reverse transcriptase inhibitors in newly diagnosed antiretroviral treatment-naive children in sub-Saharan Africa | Samples with already known results |
|  | Wiysonge, 2005 | Vaginal disinfection for preventing mother-to-child transmission of HIV infection | Review |
|  | Sone, 2017 | Prevalence and Identification of Serum Markers Associated with Vertical Transmission of Hepatitis B in Pregnant Women in Yaounde, Cameroon | No data prevalence on vertical transmission of HIV in Cameroon |
|  | Sangong, 2018 | The problem of lost to follow-up of mother-child pairs enrolled in the PMTCT program in Dschang District Hospital-Cameroon | No data prevalence on vertical transmission of HIV in Cameroon |
|  | Fokam, 2019 | Evaluation of treatment response, drug resistance and HIV-1 variability among adolescents on first- and second-line antiretroviral therapy: a study protocol for a prospective observational study in the centre region of Cameroon (EDCTP READY-study) | No data prevalence on vertical transmission of HIV in Cameroon |
|  | Villabona-Arenas, 2018 | Noninvasive western lowland gorilla's health monitoring: A decade of simian immunodeficiency virus surveillance in southern Cameroon | No data prevalence on vertical transmission of HIV in Cameroon |
|  | Uneke, 2017 | Promoting evidence informed policy making in Nigeria: a review of the maternal, newborn and child health policy development process | Review |
|  | Billong, 2017 | Feasibility Study of HIV Sentinel Surveillance using PMTCT data in Cameroon: from Scientific Success to Programmatic Failure | No data prevalence on vertical transmission of HIV in Cameroon |
|  | Atanga, 2018 | Using a composite adherence tool to assess ART response and risk factors of poor adherence in pregnant and breastfeeding HIV-positive Cameroonian women at 6 and 12 months after initiating option B | No data prevalence on vertical transmission of HIV in Cameroon |
|  | Njouom, 2005 | Low risk of mother-to-child transmission of hepatitis C virus in Yaounde, Cameroon: the ANRS 1262 study | No data prevalence on vertical transmission of HIV in Cameroon |
|  | Dionne-Odom, 2016 | Factors Associated with PMTCT Cascade Completion in Four African Countries | No data prevalence on vertical transmission of HIV in Cameroon |
|  | Dionne-Odom, 2016 | Hepatitis B, HIV, and Syphilis Seroprevalence in Pregnant Women and Blood Donors in Cameroon | No data prevalence on vertical transmission of HIV in Cameroon |
|  | Kuete, 2016 | Sexual Practices, Fertility Intentions, and Awareness to Prevent Mother-to-Child Transmission of HIV Among Infected Pregnant Women at the Yaounde Central Hospital | No data prevalence on vertical transmission of HIV in Cameroon |
|  | Stringer, 2010 | Coverage of nevirapine-based services to prevent mother-to-child HIV transmission in 4 African countries | No data prevalence on vertical transmission of HIV in Cameroon |
|  | Sanou, 2016 | Nutrition Habits and Health Outcomes of Breastfeeding HIV-positive mothers in the Dschang Health District, West Region Cameroon | No data prevalence on vertical transmission of HIV in Cameroon |
|  | Tiotsia, 2018 | Knowledge on STIs / HIV / AIDS, Stigma-Discrimination and sexual behaviors AMONG students of the University of Dschang, in Cameroon | No data prevalence on vertical transmission of HIV in Cameroon |
|  | Nkuoh, 2010 | Barriers to men's participation in antenatal and prevention of mother-to-child HIV transmission care in Cameroon, Africa | No data prevalence on vertical transmission of HIV in Cameroon |
|  | Sama, 2017 | Prevalence of maternal HIV infection and knowledge on mother-to-child transmission of HIV and its prevention among antenatal care attendees in a rural area in northwest Cameroon | No data prevalence on vertical transmission of HIV in Cameroon |
|  | Nkuoh, 2013 | Women's attitudes toward their partners' involvement in antenatal care and prevention of mother-to-child transmission of HIV in Cameroon, Africa | No data prevalence on vertical transmission of HIV in Cameroon |
|  | Nguefack, 2016 | Obstetrical, maternal characteristics and outcome of HIV-infected rapid progressor infants at Yaounde: a retrospective study | Samples with already known results |
|  | Siemieniuk, 2017 | Antiretroviral therapy in pregnant women living with HIV: a clinical practice guideline | No data prevalence on vertical transmission of HIV in Cameroon |
|  | Wiysonge, 2005 | Vitamin A supplementation for reducing the risk of mother-to-child transmission of HIV infection | Review |
|  | Kuete, 2016 | Scale up use of family planning services to prevent maternal transmission of HIV among discordant couples: a cross-sectional study within a resource-limited setting | No data prevalence on vertical transmission of HIV in Cameroon |
|  | Bigna, 2014 | Effect of mobile phone reminders on follow-up medical care of children exposed to or infected with HIV in Cameroon (MORE CARE): a multicentre, single-blind, factorial, randomised controlled trial | No data prevalence on vertical transmission of HIV in Cameroon |
|  | Loriette, 2015 | [An experience of hepatitis B control in a rural area in Far North Cameroon] | No data prevalence on vertical transmission of HIV in Cameroon |
|  | Bongajum, 2018 | An assessment of antiretroviral drug initiation to pregnant women of unknown HIV status during labour and delivery in Cameroon | No data prevalence on vertical transmission of HIV in Cameroon |
|  | Bain, 2015 | Ethical issues surrounding the provider initiated opt--Out prenatal HIV screening practice in Sub-Saharan Africa: a literature review | Review |
|  | Shey, 2002 | Vaginal disinfection during labour for reducing the risk of mother-to-child transmission of HIV infection | Review |
|  | Reinsma, 2016 | The potential effectiveness of the nutrition improvement program on infant and young child feeding and nutritional status in the Northwest and Southwest regions of Cameroon, Central Africa | No data prevalence on vertical transmission of HIV in Cameroon |
|  | Egbe, 2016 | Estimating HIV Incidence during Pregnancy and Knowledge of Prevention of Mother-to-Child Transmission with an Ad Hoc Analysis of Potential Cofactors | No data prevalence on vertical transmission of HIV in Cameroon |
|  | Orne-Gliemann, 2010 | Couple-oriented prenatal HIV counseling for HIV primary prevention: an acceptability study | No data prevalence on vertical transmission of HIV in Cameroon |
|  | Etienne, 2012 | Noninvasive follow-up of simian immunodeficiency virus infection in wild-living nonhabituated western lowland gorillas in Cameroon | No data prevalence on vertical transmission of HIV in Cameroon |
|  | Chi, 2015 | Implementation and Operational Research: Reconstructing the PMTCT Cascade Using Cross-sectional Household Survey Data: The PEARL Study | Data unclear |
|  | Freeman, 2012 | Comparison of HIV-positive women with children and without children accessing HIV care and treatment in the IeDEA Central Africa cohort | No data prevalence on vertical transmission of HIV in Cameroon |
|  | Orne-Gliemann, 2013 | Increasing HIV testing among male partners | No data prevalence on vertical transmission of HIV in Cameroon |
|  | Nakakeeto, 2009 | The global strategy to eliminate HIV infection in infants and young children: a seven-country assessment of costs and feasibility | No data prevalence on vertical transmission of HIV in Cameroon |
|  | Muko, 2004 | Preventing mother-to-child transmission: factors affecting mothers' choice of feeding--a case study from Cameroon | No data prevalence on vertical transmission of HIV in Cameroon |
|  | Turriziani, 2008 | Study of the genotypic resistant pattern in HIV-infected women and children from rural west Cameroon | No data prevalence on vertical transmission of HIV in Cameroon |
|  | Welty, 2005 | Integrating prevention of mother-to-child HIV transmission into routine antenatal care: the key to program expansion in Cameroon | No data prevalence on vertical transmission of HIV in Cameroon |
|  | Ekouevi, 2012 | Health facility characteristics and their relationship to coverage of PMTCT of HIV services across four African countries: the PEARL study | No data prevalence on vertical transmission of HIV in Cameroon |
|  | Fru, 2014 | Baseline demographic, clinical and immunological profiles of HIV-infected children at the Yaounde Gynaeco-Obstetric and Pediatric hospital, Cameroon | Samples with already known results |
|  | Atanga, 2017 | Retention in care and reasons for discontinuation of lifelong antiretroviral therapy in a cohort of Cameroonian pregnant and breastfeeding HIV-positive women initiating 'Option B+' in the South West Region | No data prevalence on vertical transmission of HIV in Cameroon |
|  | Fokam, 2018 | Next-generation sequencing provides an added value in determining drug resistance and viral tropism in Cameroonian HIV-1 vertically infected children | Samples with already known results |
|  | Shey, 2002 | Vitamin A supplementation for reducing the risk of mother-to-child transmission of HIV infection | Review |
|  | Divaris, 2012 | Adult HIV care resources, management practices and patient characteristics in the Phase 1 IeDEA Central Africa cohort | No data prevalence on vertical transmission of HIV in Cameroon |
|  | Desclaux, 2009 | Counseling and choosing between infant-feeding options: overall limits and local interpretations by health care providers and women living with HIV in resource-poor countries (Burkina Faso, Cambodia, Cameroon) | No data prevalence on vertical transmission of HIV in Cameroon |
|  | Aghokeng, 2013 | Virological outcome and patterns of HIV-1 drug resistance in patients with 36 months' antiretroviral therapy experience in Cameroon | No data prevalence on vertical transmission of HIV in Cameroon |
|  | Kfutwah, 2013 | An antiretroviral drug-naïve human immunodeficiency virus-1 infected woman with a persistent non-reactive proviral deoxyribonucleic acid polymerase chain reaction: a case report | No data prevalence on vertical transmission of HIV in Cameroon |
|  | Ndawinz, 2013 | Factors associated with late antiretroviral therapy initiation in Cameroon: a representative multilevel analysis | No data prevalence on vertical transmission of HIV in Cameroon |
|  | Jashi, 2013 | Informing policy and programme decisions for scaling up the PMTCT and paediatric HIV response through joint technical missions | No data prevalence on vertical transmission of HIV in Cameroon |
|  | Mutarambirwa, 2017 | Term Abdominal Pregnancy Revealed by Amnioperitoneum in Rural Area | No data prevalence on vertical transmission of HIV in Cameroon |
|  | Tita, 2006 | Factors associated with the awareness and practice of evidence-based obstetric care in an African setting | No data prevalence on vertical transmission of HIV in Cameroon |
|  | Bigna, 2014 | Factors associated with non-adherence to scheduled medical follow-up appointments among Cameroonian children requiring HIV care: a case-control analysis of the usual-care group in the MORE CARE trial | Samples with already known results |
|  | Menu, 1997 | Insights into the mechanisms of vertical transmission of HIV-1. BIOMED2 Working Group on the in utero transmission of HIV-1 | No data prevalence on vertical transmission of HIV in Cameroon |
|  | NR, 1996 | Conclusions of a round-table that took place during a seminar on the prevention of TB and HIV transmission in health care settings, Douala, Cameroon, January 1995 | No data prevalence on vertical transmission of HIV in Cameroon |
|  | Kfutwah, 2006 | Tumour necrosis factor-alpha stimulates HIV-1 replication in single-cycle infection of human term placental villi fragments in a time, viral dose and envelope dependent mNjomr | No data prevalence on vertical transmission of HIV in Cameroon |
|  | Njom, 2011 | Virological profile of pregnant HIV positive women with high levels of CD4 count in low income settings: can viral load help as eligibility criteria for maternal triple ARV prophylaxis (WHO 2010 option B)? | No data prevalence on vertical transmission of HIV in Cameroon |
|  | Stringer, 2013 | Measuring coverage in MNCH: population HIV-free survival among children under two years of age in four African countries | Data unclear |
|  | Kfutwah, 2009 | Plasmodium falciparum infection significantly impairs placental cytokine profile in HIV infected Cameroonian women | No data prevalence on vertical transmission of HIV in Cameroon |
|  | Egbe, 2017 | Cesarean delivery technique among HIV positive women with sub-optimal antenatal care uptake at the Douala General Hospital, Cameroon: case series report | Sample size<10 participants |
|  | Chaix-Baudier, 1998 | First case of mother-to-infant HIV type 1 group O transmission and evolution of C2V3 sequences in the infected child. French HIV Pediatric Cohort Study Group | Case report |
|  | Skalsky, 1996 | [Role of hepatotropic viruses in liver pathology in Southwestern Cameroon] | No data prevalence on vertical transmission of HIV in Cameroon |
|  | Boeke, 2021 | Point-of-care testing can achieve same-day diagnosis for infants and rapid ART initiation: results from government programmes across six African countries | Data unclear |
|  | Abange, 2021 | Alteration of the gut fecal microbiome in children living with HIV on antiretroviral therapy in Yaounde, Cameroon | Samples with already known results |
|  | Fomulu, 2009 | Efficacy of highly active triple antiretroviral therapy in preventing mother-to-child HIV transmission in the university teaching hospitals in Yaounde, Cameroon | No data prevalence on vertical transmission of HIV in Cameroon |
|  | Awuba, 2008 | HIV/AIDS in Cameroon: Rising gender issues in policy-making matters. | No data prevalence on vertical transmission of HIV in Cameroon |
|  | Amenu, 2015 | Malaria and HIV/AIDS Co-infections | No data prevalence on vertical transmission of HIV in Cameroon |
|  | Tanjong, 2016 | Sero-prevalence of Human Immunodeficiency Virus and hepatitis viruses and their correlation with CD4 T-cell lymphocyte counts in pregnant women in the Buea Health District of Cameroon | No data prevalence on vertical transmission of HIV in Cameroon |
|  | Bwana, 2016 | Accessibility of services for early infant diagnosis of Human Immunodeficiency Virus in sub-Saharan Africa: a systematic review | systematic review |
|  | Bongajum, 2018 | An assessment of antiretroviral drug initiation to pregnant women of unknown HIV status during labour and delivery in Cameroon | No data prevalence on vertical transmission of HIV in Cameroon |
|  | Hamela, 2014 | Evaluating the Benefits of Incorporating Traditional Birth Attendants in HIV Prevention of Mother to Child Transmission Service Delivery in Lilongwe, Malawi | No data prevalence on vertical transmission of HIV in Cameroon |
|  | Temitope, 2017 | Health Workers' Knowledge of Preventing Mother-To-Child Transmission of HIV in Benin City, Edo State, Nigeria | No data prevalence on vertical transmission of HIV in Cameroon |
|  | American Academy of Pediatrics Committee on Pediatrics AIDS | Disclosure of illness status to children and adolescents with HIV infection | No data prevalence on vertical transmission of HIV in Cameroon |
|  | Francisca, 2011 | HIV exposure and related newborn morbidity and mortality in the University Teaching Hospital of Yaoundé, Cameroon | No data prevalence on vertical transmission of HIV in Cameroon |
|  | Manji | The use of Anti-Retroviral Drugs in the prevention of Mother To Child Transmission (PMTCT) of HIV Review Paper | Review |
|  | Ugoeze, 2016 | “Comparison of performance of prevention of mother-to-child transmission (PMTCT) of HIV/AIDS cascade between public and private health facilities in a community PMTCT implementation in north central Nigeria.” | No data prevalence on vertical transmission of HIV in Cameroon |
|  | Teclebirhan, 2010 | Prevention of mother to child transmission of HIV/AIDS in Eritrea: the Eritrean experience. | No data prevalence on vertical transmission of HIV in Cameroon |
|  | Teasdale, 2008 | Enhancing PMTCT programmes through psychosocial support and empowerment of women: The Mothers2mothers model of care. | No data prevalence on vertical transmission of HIV in Cameroon |
|  | Djuidje, 2015 | Human Immunodeficiency Virus and Hepatitis C Virus Co-infection in Cameroon: Investigation of the Genetic Diversity and Virulent Circulating Strains. | No data prevalence on vertical transmission of HIV in Cameroon |
|  | Andreas, 2012 | The frequency and magnitude of growth failure in a group of HIV-infected children in Cameroon. | No data prevalence on vertical transmission of HIV in Cameroon |
|  | Isah, 2014 | Institutional Profile of PHC Facilities in Nigeria and its Implication for PMTCT Scale-Up and Decentralization of HIV and AIDS Services: Nasarawa State as a Case Study | No data prevalence on vertical transmission of HIV in Cameroon |
|  | Mkoma, 2012 | Improving Child survival through enhancing Prevention of Mother to Child Transmission of HIV. | No data prevalence on vertical transmission of HIV in Cameroon |
|  | Nsojo, 2010 | Comparative evaluation of Amplicor HIV-1 DNA test, version 1.5, by manual and automated DNA extraction methods using venous blood and dried blood spots for HIV-1 DNA PCR testing. | No data prevalence on vertical transmission of HIV in Cameroon |
|  | Igumbor, 2006 | Effect of exposure to clinic-based health education interventions on behavioural intention to prevent mother-to-child transmission of HIV infection. | No data prevalence on vertical transmission of HIV in Cameroon |
|  | Otieno, 2018 | KNOWLEDGE AND ATTITUDE AS DETERMINANT FACTORS IN HIV CARE AMONG PREGNANT WOMEN IN RACHUONYO NORTH, HOMA-BAY COUNTY, KENYA. | No data prevalence on vertical transmission of HIV in Cameroon |
|  | Nduati, 2019 | Roll-out of prevention of mother-to-child transmission of Human Immunodeficiency Virus in rural Kenya | No data prevalence on vertical transmission of HIV in Cameroon |
|  | Lejeng, 2020 | Mothers’ knowledge of mother-to-child transmission of HIV and infant feeding practices in Juba, South Sudan. | No data prevalence on vertical transmission of HIV in Cameroon |
|  | Negash, 2018 | Women's utilisation of prevention of mother-to-child transmission of human immunodeficiency virus services in Addis Ababa, Ethiopia. Health SA. | No data prevalence on vertical transmission of HIV in Cameroon |
|  | King, 2020 | PMTCT Option B+ 2012 to 2018 — Taking stock: barriers and strategies to improve adherence to Option B+ in urban and rural Uganda. | No data prevalence on vertical transmission of HIV in Cameroon |
|  | Samuels, 2014 | Foeto-maternal outcome of HIV-positive pregnant women on Highly Active Antiretroviral Therapy. | No data prevalence on vertical transmission of HIV in Cameroon |
|  | Douaguibe, 2017 | Suivi de la femme enceinte seropositive au VIH a l'Hopital de be a Lome | No data prevalence on vertical transmission of HIV in Cameroon |
|  | Azouma, 2012 | Prevention de la transmission mere enfant du VIH/SIDA au Chu de Kara (Togo) : Bilan de l’evolution sur quatre ans d’activite | No data prevalence on vertical transmission of HIV in Cameroon |
|  | Otieno, 2017 | Socio-cultural Factors Influencing Utilization of Prevention-of-Mother-to-Child-Transmission of HIV Strategies among Women Attending Antenatal Care Clinics in Rachuonyo North Sub-County-Homa-Bay County | No data prevalence on vertical transmission of HIV in Cameroon |
|  | Akani, 2006 | HIV sero-discordance among Nigerian couples: challenges and controversies | No data prevalence on vertical transmission of HIV in Cameroon |
|  | KATLAMA, 1989 | Aspects epidemiologiques, cliniques et therapeutiques de l'infection a VIH.. | No data prevalence on vertical transmission of HIV in Cameroon |
|  | Takassi, 2020 | Partage du statut serologique au sein des couples serodiscordants dans un protocole de PTME en pediatrie au CHU Sylvanus Olympio (Togo) | No data prevalence on vertical transmission of HIV in Cameroon |
|  | King, 2018 | PMTCT Option B+ 2012 to 2018 - Taking stock: barriers and strategies to improve adherence to Option B+ in urban and rural Uganda | No data prevalence on vertical transmission of HIV in Cameroon |
|  | Isah, 2014 | Institutional Profile of PHC Facilities in Nigeria and its Implication for PMTCT Scale-Up and Decentralization of HIV and AIDS Services: Nasarawa State as a Case Study. | No data prevalence on vertical transmission of HIV in Cameroon |
|  | Djadou, 2012 | Connaissances, attitudes et pratiques en focus group des clients des sites de prévention de la transmission du VIH de la mère à son enfant au Togo en 2010 | No data prevalence on vertical transmission of HIV in Cameroon |
|  | Nduati, 2019 | Roll-out of prevention of mother-to-child transmission of Human Immunodeficiency Virus in rural Kenya | No data prevalence on vertical transmission of HIV in Cameroon |
|  | Nguhiu, 2017 | ROLE OF MATERNAL, PSYCHOSOCIAL AND SOCIAL-CULTURAL FACTORS IN HIV-EXPOSED INFANTS' SERVICE UPTAKE | No data prevalence on vertical transmission of HIV in Cameroon |
|  | Chiabi, 2006 | DISCLOSURE OF HIV ILLNESS STATUS TO CHILDREN AND ADOLESCENTS: When, how and by who? | Brief communication |
|  | Esemu, 2019 | Impact of HIV-1 infection on the IGF-1 axis and angiogenic factors in pregnant Cameroonian women receiving antiretroviral therapy | Data with outliers |
|  | Penda, 2018 | Feasibility and utility of active case finding of HIV-infected children and adolescents by provider-initiated testing and counselling: evidence from the Laquintinie hospital in Douala, Cameroon. | Data with outliers |
|  | Yumo, 2021 | Human immunodeficiency virus case detection and antiretroviral therapy enrollment among children below and above 18 months old: A comparative analysis from Cameroon. | Data with outliers |
|  | Mkoma, 2012 | Improving Child survival through enhancing Prevention of Mother to Child Transmission of HIV. | No data prevalence on vertical transmission of HIV in Cameroon |
|  | Aregbesola, 2018 | Self-efficacy and antiretroviral therapy adherence among HIV positive pregnant women in South-West Nigeria: a mixed methods study. | No data prevalence on vertical transmission of HIV in Cameroon |
|  | Negash, 2018 | Women’s utilisation of prevention of mother-to-child transmission of human immunodeficiency virus services in Addis Ababa, Ethiopia. | No data prevalence on vertical transmission of HIV in Cameroon |
|  | Irinyenikan, 2019 | Knowledge and practice of HIV testing for PMTCT among antenatal clinic attendees | No data prevalence on vertical transmission of HIV in Cameroon |

**References of excluded studies**:

1. Rao A, Schwartz S, Billong SC, Bowring A, Fouda G, Ndonko F, Njindam I, Levitt D, Bissek AC, Njoya O, Baral S. Predictors of early childhood HIV testing among children of sex workers living with HIV in Cameroon. BMC Public Health. 2019 May 29;19(Suppl 1):602. doi: 10.1186/s12889-019-6812-3. PMID: 31138289; PMCID: PMC6538542.
2. Sama CB, Feteh VF, Tindong M, Tanyi JT, Bihle NM, Angwafo FF 3rd. Prevalence of maternal HIV infection and knowledge on mother-to-child transmission of HIV and its prevention among antenatal care attendees in a rural area in northwest Cameroon. PLoS One. 2017 Feb 15;12(2):e0172102. doi: 10.1371/journal.pone.0172102. PMID: 28199373; PMCID: PMC5310783.
3. Yah CS, Tambo E. Why is mother to child transmission (MTCT) of HIV a continual threat to new-borns in sub-Saharan Africa (SSA). J Infect Public Health. 2019 Mar-Apr;12(2):213-223. doi: 10.1016/j.jiph.2018.10.008. Epub 2018 Nov 8. PMID: 30415979.
4. Ghoma Linguissi LS, Sagna T, Soubeiga ST, Gwom LC, Nkenfou CN, Obiri-Yeboah D, Ouattara AK, Pietra V, Simpore J. Prevention of mother-to-child transmission (PMTCT) of HIV: a review of the achievements and challenges in Burkina-Faso. HIV AIDS (Auckl). 2019 Jul 24;11:165-177. doi: 10.2147/HIV.S204661. PMID: 31440104; PMCID: PMC6664853.
5. Penda CI, Ndongo FA, Bissek AZ, Téjiokem MC, Sofeu C, Moukoko Eboumbou EC, Mindjouli S, Desmonde S, Njock LR. Practices of Care to HIV-Infected Children: Current Situation in Cameroon. Clin Med Insights Pediatr. 2019 May 3;13:1179556519846110. doi: 10.1177/1179556519846110. PMID: 31105436; PMCID: PMC6501467.
6. Budhwani H, Hearld KR, Dionne-Odom J, Manga S, Nulah K, Khan M, Welty T, Welty E, Tita AT. HIV Status and Contraceptive Utilization among Women in Cameroon. J Int Assoc Provid AIDS Care. 2019 Jan-Dec;18:2325958219826596. doi: 10.1177/2325958219826596. PMID: 30776955; PMCID: PMC6748529.
7. Ngoufack MN, Nkenfou CN, Tiedeu BA, Nguefack-Tsague G, Mouafo LCM, Dambaya B, Nguefeu CN, Ndzi EN, Billong SC, Mbacham WF, Ndjolo A. CCR2 polymorphism and HIV: mutation in both mother and child is associated with higher transmission. Int J Biochem Mol Biol. 2019 Oct 15;10(4):42-48. PMID: 31777682; PMCID: PMC6874776.
8. Ford CE, Coetzee D, Winston J, Chibwesha CJ, Ekouevi DK, Welty TK, Tih PM, Maman S, Stringer EM, Stringer JSA, Chi BH. Maternal Decision-Making and Uptake of Health Services for the Prevention of Mother-to-Child HIV Transmission: A Secondary Analysis. Matern Child Health J. 2019 Jan;23(1):30-38. doi: 10.1007/s10995-018-2588-9. PMID: 30022401.
9. Ikomey GM, Assoumou MCO, Gichana JO, Njenda D, Mikasi SG, Mesembe M, Lyonga E, Jacobs GB. Observed HIV drug resistance associated mutations amongst naïve immunocompetent children in Yaoundé, Cameroon. Germs. 2017 Dec 5;7(4):178-185. doi: 10.18683/germs.2017.1124. PMID: 29264355; PMCID: PMC5734927.
10. Landefeld CC, Fomenou LA, Ateba F, Msellati P. Prevention of Mother-to-Child Transmission of HIV in Yaounde: Barrier to Care. AIDS Care. 2018 Jan;30(1):116-120. doi: 10.1080/09540121.2017.1390540. Epub 2017 Oct 16. PMID: 29034724.
11. Biccard BM, Madiba TE, Kluyts HL, Munlemvo DM, Madzimbamuto FD, Basenero A, Gordon CS, Youssouf C, Rakotoarison SR, Gobin V, Samateh AL, Sani CM, Omigbodun AO, Amanor-Boadu SD, Tumukunde JT, Esterhuizen TM, Manach YL, Forget P, Elkhogia AM, Mehyaoui RM, Zoumeno E, Ndayisaba G, Ndasi H, Ndonga AKN, Ngumi ZWW, Patel UP, Ashebir DZ, Antwi-Kusi AAK, Mbwele B, Sama HD, Elfiky M, Fawzy MA, Pearse RM; African Surgical Outcomes Study (ASOS) investigators. Perioperative patient outcomes in the African Surgical Outcomes Study: a 7-day prospective observational cohort study. Lancet. 2018 Apr 21;391(10130):1589-1598. doi: 10.1016/S0140-6736(18)30001-1. Epub 2018 Jan 3. PMID: 29306587.
12. Kufe NC, Metekoua C, Nelly M, Tumasang F, Mbu ER. Retention of health care workers at health facility, trends in the retention of knowledge and correlates at 3rd year following training of health care workers on the prevention of mother-to-child transmission (PMTCT) of HIV-National Assessment. BMC Health Serv Res. 2019 Jan 29;19(1):78. doi: 10.1186/s12913-019-3925-4. PMID: 30696489; PMCID: PMC6352341.
13. Awungafac G, Njukeng PA, Ndasi JA, Mbuagbaw LT. Prevention of mother-to-child transmission of the Human Immunodeficiency Virus: investigating the uptake and utilization of maternal and child health services in Tiko health district, Cameroon. Pan Afr Med J. 2015 Jan 7;20:20. doi: 10.11604/pamj.2015.20.20.5137. PMID: 25995817; PMCID: PMC4431405.
14. Mouafo LCM, Dambaya B, Ngoufack NN, Nkenfou CN. Host Molecular Factors and Viral Genotypes in the Mother-to-Child HIV-1 Transmission in Sub-Saharan Africa. J Public Health Afr. 2017 Jul 3;8(1):594. doi: 10.4081/jphia.2017.594. PMID: 28748061; PMCID: PMC5510234.
15. Chersich MF, Newbatt E, Ng'oma K, de Zoysa I. UNICEF's contribution to the adoption and implementation of option B+ for preventing mother-to-child transmission of HIV: a policy analysis. Global Health. 2018 Jun 1;14(1):55. doi: 10.1186/s12992-018-0369-2. PMID: 29859098; PMCID: PMC5984744.
16. Bianchi F, Clemens S, Arif Z, Sacks E, Cohn J; EGPAF POC EID Study Team. Acceptability of Routine Point-of-Care Early Infant Diagnosis in Eight African Countries: Findings From a Qualitative Assessment of Clinical and Laboratory Personnel. J Acquir Immune Defic Syndr. 2020 Jul 1;84 Suppl 1:S41-S48. doi: 10.1097/QAI.0000000000002372. PMID: 32520914.
17. Mekue LM, Nkenfou CN, Ndukong E, Yatchou L, Dambaya B, Ngoufack MN, Kameni JK, Kuiaté JR, Ndjolo A. HLA A*32 is associated to HIV acquisition while B*44 and B*53 are associated with protection against HIV acquisition in perinatally exposed infants. BMC Pediatr. 2019 Jul 23;19(1):249. doi: 10.1186/s12887-019-1620-6. PMID: 31337377; PMCID: PMC6647251.
18. Bishop D, Dyer RA, Maswime S, Rodseth RN, van Dyk D, Kluyts HL, Tumukunde JT, Madzimbamuto FD, Elkhogia AM, Ndonga AKN, Ngumi ZWW, Omigbodun AO, Amanor-Boadu SD, Zoumenou E, Basenero A, Munlemvo DM, Youssouf C, Ndayisaba G, Antwi-Kusi A, Gobin V, Forget P, Mbwele B, Ndasi H, Rakotoarison SR, Samateh AL, Mehyaoui R, Patel-Mujajati U, Sani CM, Esterhuizen TM, Madiba TE, Pearse RM, Biccard BM; ASOS investigators. Maternal and neonatal outcomes after caesarean delivery in the African Surgical Outcomes Study: a 7-day prospective observational cohort study. Lancet Glob Health. 2019 Apr;7(4):e513-e522. doi: 10.1016/S2214-109X(19)30036-1. Erratum in: Lancet Glob Health. 2019 Aug;7(8):e1019. PMID: 30879511.
19. Dambaya B, Nkenfou CN, Ambada G, Ikomey GM, Mouafo LM, Ngoufack N, Ndzi EN, Této G, Nanfack A, Sonela N, Fokam J, Flobert N, Colizzi V, Ndjolo A. Differential expression of Fas receptors (CD95) and Fas ligands (CD95L) in HIV infected and exposed uninfected children in Cameroon versus unexposed children. Pan Afr Med J. 2019 Sep 18;34:39. doi: 10.11604/pamj.2019.34.39.15038. PMID: 31762906; PMCID: PMC6859037.
20. Liotta G, Marazzi MC, Mothibi KE, Zimba I, Amangoua EE, Bonje EK, Bossiky BN, Robinson PA, Scarcella P, Musokotwane K, Palombi L, Germano P, Narciso P, de Luca A, Alumando E, Mamary SH, Magid NA, Guidotti G, Mancinelli S, Orlando S, Peroni M, Buonomo E, Nielsen-Saines K. Elimination of Mother-To-Child Transmission of HIV Infection: The Drug Resource Enhancement against AIDS and Malnutrition Model. Int J Environ Res Public Health. 2015 Oct 21;12(10):13224-39. doi: 10.3390/ijerph121013224. PMID: 26506365; PMCID: PMC4627027.
21. Luo C, Hirnschall G, Rodrigues J, Romano S, Essajee S, Rogers B, McCarthy E, Mwango A, Sangrujee N, Adler MR, Houston JC, Langa JO, Urso M, Bolu O, Tene G, Elat Nfetam JB, Kembou E, Phelps BR. Translating Technical Support Into Country Action: The Role of the Interagency Task Team on the Prevention and Treatment of HIV Infection in Pregnant Women, Mothers, and Children in the Global Plan Era. J Acquir Immune Defic Syndr. 2017 May 1;75 Suppl 1:S7-S16. doi: 10.1097/QAI.0000000000001332. PMID: 28398992.
22. Napierala Mavedzenge S, Newman JE, Nduwimana M, Bukuru H, Kariyo P, Niyongabo T, Mbaya M, Mukumbi H, Kamgaing N, Obama MT, Akam W, Atibu J, Kiumbu M, Hemingway-Foday J. HIV infection among children and adolescents in Burundi, Cameroon, and the Democratic Republic of Congo. AIDS Care. 2017 Aug;29(8):1026-1033. doi: 10.1080/09540121.2016.1273472. Epub 2017 Jan 9. PMID: 28064538.
23. Tantchou J, Tijou-Traoré A. The mother's *carnet de santé* (health booklet) in Cameroon: a tool for preventing mother-to-child transmission of HIV?. Anthropol Med. 2019 Apr;26(1):104-118. doi: 10.1080/13648470.2017.1334039. Epub 2018 Feb 27. PMID: 29482337.
24. Nkwabong E, Meboulou Nguel R, Kamgaing N, Keddi Jippe AS. Knowledge, attitudes and practices of health personnel of maternities in the prevention of mother-to-child transmission of HIV in a sub-Saharan African region with high transmission rate: some solutions proposed. BMC Pregnancy Childbirth. 2018 Jun 14;18(1):227. doi: 10.1186/s12884-018-1876-0. PMID: 29898688; PMCID: PMC6000955.
25. Labhardt ND, Manga E, Ndam M, Balo JR, Bischoff A, Stoll B. Early assessment of the implementation of a national programme for the prevention of mother-to-child transmission of HIV in Cameroon and the effects of staff training: a survey in 70 rural health care facilities. Trop Med Int Health. 2009 Mar;14(3):288-93. doi: 10.1111/j.1365-3156.2009.02221.x. Epub 2009 Jan 28. PMID: 19187522.
26. Luma HN, Jua P, Donfack OT, Kamdem F, Ngouadjeu E, Mbatchou HB, Doualla MS, Mapoure YN. Late presentation to HIV/AIDS care at the Douala general hospital, Cameroon: its associated factors, and consequences. BMC Infect Dis. 2018 Jul 3;18(1):298. doi: 10.1186/s12879-018-3204-8. PMID: 29970017; PMCID: PMC6029364.
27. Kalua T, Tippett Barr BA, van Oosterhout JJ, Mbori-Ngacha D, Schouten EJ, Gupta S, Sande A, Zomba G, Tweya H, Lungu E, Kajoka D, Tih P, Jahn A. Lessons Learned From Option B+ in the Evolution Toward "Test and Start" From Malawi, Cameroon, and the United Republic of Tanzania. J Acquir Immune Defic Syndr. 2017 May 1;75 Suppl 1(Suppl 1):S43-S50. doi: 10.1097/QAI.0000000000001326. PMID: 28398996; PMCID: PMC6415299.
28. Bianchi F, Cohn J, Sacks E, Bailey R, Lemaire JF, Machekano R; EGPAF POC EID Study Team. Evaluation of a routine point-of-care intervention for early infant diagnosis of HIV: an observational study in eight African countries. Lancet HIV. 2019 Jun;6(6):e373-e381. doi: 10.1016/S2352-3018(19)30033-5. Epub 2019 Apr 12. PMID: 30987937.
29. Zoung-Kanyi Bissek AC, Yakana IE, Monebenimp F, Chaby G, Akondeng L, Angwafor SA, Lok C, Njamnshi AK, Muna WF. Knowledge of Pregnant Women on Mother-to-Child Transmission of HIV in Yaoundé. Open AIDS J. 2011;5:25-8. doi: 10.2174/1874613601105010025. Epub 2011 Mar 18. PMID: 21643423; PMCID: PMC3103904.
30. Torimiro JN, Nanfack A, Takang W, Keou CK, Joyce AN, Njefi K, Agyingi K, Domkam I, Takou D, Moudourou S, Sosso S, Mbu RE. Rates of HBV, HCV, HDV and HIV type 1 among pregnant women and HIV type 1 drug resistance-associated mutations in breastfeeding women on antiretroviral therapy. BMC Pregnancy Childbirth. 2018 Dec 22;18(1):504. doi: 10.1186/s12884-018-2120-7. PMID: 30577760; PMCID: PMC6303885.
31. Inzaule SC, Jordan MR, Bello G, Wadonda-Kabondo N, Mounerou S, Mbulli IA, Akanmu SA, Vubil A, Hunt G, Kaleebu P, Mthethwa-Hleza S, Dzangare J, Njukeng P, Penazzato M, Rinke de Wit TF, Eshleman SH, Bertagnolio S; Infant HIV Drug Resistance Survey Team. High levels of resistance to nucleoside/nucleotide reverse transcriptase inhibitors in newly diagnosed antiretroviral treatment-naive children in sub-Saharan Africa. AIDS. 2020 Aug 1;34(10):1567-1570. doi: 10.1097/QAD.0000000000002580. PMID: 32443062.
32. Wiysonge CS, Shey MS, Shang JD, Sterne JA, Brocklehurst P. Vaginal disinfection for preventing mother-to-child transmission of HIV infection. Cochrane Database Syst Rev. 2005 Oct 19;(4):CD003651. doi: 10.1002/14651858.CD003651.pub2. PMID: 16235334.
33. Sone LHE, Voufo RA, Dimodi HT, Kengne M, Gueguim C, Ngah N, Oben J, Ngondi JL. Prevalence and Identification of Serum Markers Associated with Vertical Transmission of Hepatitis B in Pregnant Women in Yaounde, Cameroon. Int J MCH AIDS. 2017;6(1):69-74. doi: 10.21106/IJMA.174. PMID: 28798895; PMCID: PMC5547227.
34. Efeutmecheh Sangong R, Tiotsia Tsapi A, Djeunang Dongho GB, Fokam J, Azeufack Ngueko Y, Bell Pallawo I, Zogning Makemjio E, Jagni Semengue EN, Bouting Mayaka G, Azetsop J, Ercoli L, Colizzi V, Panà A, Russo G, Sanou Sobze M. The problem of lost to follow-up of mother-child pairs enrolled in the PMTCT program in Dschang District Hospital-Cameroon. Ig Sanita Pubbl. 2018 Jul-Aug;74(4):337-347. PMID: 30767949.
35. Fokam J, Santoro MM, Takou D, Njom-Nlend AE, Ndombo PK, Kamgaing N, Kamta C, Essiane A, Sosso SM, Ndjolo A, Colizzi V, Perno CF. Evaluation of treatment response, drug resistance and HIV-1 variability among adolescents on first- and second-line antiretroviral therapy: a study protocol for a prospective observational study in the centre region of Cameroon (EDCTP READY-study). BMC Pediatr. 2019 Jul 5;19(1):226. doi: 10.1186/s12887-019-1599-z. PMID: 31277610; PMCID: PMC6612130.
36. Villabona-Arenas CJ, Ayouba A, Esteban A, D'arc M, Mpoudi Ngole E, Peeters M. Noninvasive western lowland gorilla's health monitoring: A decade of simian immunodeficiency virus surveillance in southern Cameroon. Ecol Evol. 2018 Oct 25;8(22):10698-10710. doi: 10.1002/ece3.4478. PMID: 30519399; PMCID: PMC6262910.
37. Uneke CJ, Sombie I, Keita N, Lokossou V, Johnson E, Ongolo-Zogo P, Uro-Chukwu HC. Promoting evidence informed policy making in Nigeria: a review of the maternal, newborn and child health policy development process. Health Promot Perspect. 2017 Sep 26;7(4):181-189. doi: 10.15171/hpp.2017.33. PMID: 29085794; PMCID: PMC5647352.
38. Billong SC, Dee J, Fokam J, Nguefack-Tsague G, Ekali GL, Fodjo R, Temgoua ES, Billong EJ, Sosso SM, Mosoko JJ, Monebenimp F, Ndjolo A, Bissek AZ, Bolu O, Elat JN. Feasibility Study of HIV Sentinel Surveillance using PMTCT data in Cameroon: from Scientific Success to Programmatic Failure. BMC Infect Dis. 2017 Jan 3;17(1):3. doi: 10.1186/s12879-016-2119-5. PMID: 28049451; PMCID: PMC5209823.
39. Atanga PN, Ndetan HT, Fon PN, Meriki HD, Muffih TP, Achidi EA, Hoelscher M, Kroidl A. Using a composite adherence tool to assess ART response and risk factors of poor adherence in pregnant and breastfeeding HIV-positive Cameroonian women at 6 and 12 months after initiating option B. BMC Pregnancy Childbirth. 2018 Oct 25;18(1):418. doi: 10.1186/s12884-018-2058-9. PMID: 30359239; PMCID: PMC6202832.
40. Njouom R, Pasquier C, Ayouba A, Tejiokem MC, Vessiere A, Mfoupouendoun J, Tene G, Eteki N, Lobe MM, Izopet J, Nerrienet E. Low risk of mother-to-child transmission of hepatitis C virus in Yaounde, Cameroon: the ANRS 1262 study. Am J Trop Med Hyg. 2005 Aug;73(2):460-6. PMID: 16103623.
41. Dionne-Odom J, Welty TK, Westfall AO, Chi BH, Ekouevi DK, Kasaro M, Tih PM, Tita AT. Factors Associated with PMTCT Cascade Completion in Four African Countries. AIDS Res Treat. 2016;2016:2403936. doi: 10.1155/2016/2403936. Epub 2016 Oct 31. PMID: 27872760; PMCID: PMC5107823.
42. Dionne-Odom J, Mbah R, Rembert NJ, Tancho S, Halle-Ekane GE, Enah C, Welty TK, Tih PM, Tita AT. Hepatitis B, HIV, and Syphilis Seroprevalence in Pregnant Women and Blood Donors in Cameroon. Infect Dis Obstet Gynecol. 2016;2016:4359401. doi: 10.1155/2016/4359401. Epub 2016 Aug 8. PMID: 27578957; PMCID: PMC4992796.
43. Kuete M, Yuan H, He Q, Tchoua Kemayou AL, Ndognjem TP, Yang F, Hu Z, Tian B, Zhao K, Zhang H, Xiong C. Sexual Practices, Fertility Intentions, and Awareness to Prevent Mother-to-Child Transmission of HIV Among Infected Pregnant Women at the Yaounde Central Hospital. Sex Med. 2016 Jun;4(2):e95-e103. doi: 10.1016/j.esxm.2016.01.004. Epub 2016 Mar 19. PMID: 27006318; PMCID: PMC5005303.
44. Stringer EM, Ekouevi DK, Coetzee D, Tih PM, Creek TL, Stinson K, Giganti MJ, Welty TK, Chintu N, Chi BH, Wilfert CM, Shaffer N, Dabis F, Stringer JS; PEARL Study Team. Coverage of nevirapine-based services to prevent mother-to-child HIV transmission in 4 African countries. JAMA. 2010 Jul 21;304(3):293-302. doi: 10.1001/jama.2010.990. PMID: 20639563.
45. Sanou Sobze M, Kien-Atsu T, Djeunang Dongho B, Fotso JR, TiotsiaTsapi A, Azeufack Ngueko Y, Ben Bechir S, Pana A, Ercoli L, Colizzi V, Russo G. Nutrition Habits and Health Outcomes of Breastfeeding HIV-positive mothers in the Dschang Health District, West Region Cameroon. Ig Sanita Pubbl. 2016 Sep-Oct;72(5):417-427. PMID: 28068675.
46. Tiotsia Tsapi A, Djeunang Dongho GB, Efeutmecheh Sangong R, Zogning Makemjio E, Jagni Semengue EN, Bell Pallawo I, Defo Tamgno E, Bita Izacar AG, Azeufack Ngueko Y, Ercoli L, Panà A, Vincenzo C, Ndoungue M, Russo G, Sanou Sobze M. Knowledge on STIs / HIV / AIDS, Stigma-Discrimination and sexual behaviors AMONG students of the University of Dschang, in Cameroon. Ig Sanita Pubbl. 2018 Sep-Oct;74(5):419-432. PMID: 30780156.
47. Nkuoh GN, Meyer DJ, Tih PM, Nkfusai J. Barriers to men's participation in antenatal and prevention of mother-to-child HIV transmission care in Cameroon, Africa. J Midwifery Womens Health. 2010 Jul-Aug;55(4):363-9. doi: 10.1016/j.jmwh.2010.02.009. PMID: 20630363.
48. Sama CB, Feteh VF, Tindong M, Tanyi JT, Bihle NM, Angwafo FF 3rd. Prevalence of maternal HIV infection and knowledge on mother-to-child transmission of HIV and its prevention among antenatal care attendees in a rural area in northwest Cameroon. PLoS One. 2017 Feb 15;12(2):e0172102. doi: 10.1371/journal.pone.0172102. PMID: 28199373; PMCID: PMC5310783.
49. Nkuoh GN, Meyer DJ, Nshom EM. Women's attitudes toward their partners' involvement in antenatal care and prevention of mother-to-child transmission of HIV in Cameroon, Africa. J Midwifery Womens Health. 2013 Jan-Feb;58(1):83-91. doi: 10.1111/j.1542-2011.2012.00208.x. PMID: 23374493.
50. Nguefack F, Dongmo R, Touffic Othman CL, Tatah S, Njiki Kinkela MN, Koki Ndombo PO. Obstetrical, maternal characteristics and outcome of HIV-infected rapid progressor infants at Yaounde: a retrospective study. Transl Pediatr. 2016 Apr;5(2):46-54. doi: 10.21037/tp.2016.04.04. PMID: 27186521; PMCID: PMC4855195.
51. Siemieniuk RAC, Lytvyn L, Mah Ming J, Mullen RM, Anam F, Otieno T, Guyatt GH, Taylor GP, Beltrán-Arroyave C, Okwen PM, Nduati R, Kinuthia J, Luma HN, Kirpalani H, Merglen A, Lesi OA, Vandvik PO, Agoritsas T, Bewley S. Antiretroviral therapy in pregnant women living with HIV: a clinical practice guideline. BMJ. 2017 Sep 11;358:j3961. doi: 10.1136/bmj.j3961. PMID: 28893728; PMCID: PMC5590100.
52. Wiysonge CS, Shey M, Kongnyuy EJ, Sterne JA, Brocklehurst P. Vitamin A supplementation for reducing the risk of mother-to-child transmission of HIV infection. Cochrane Database Syst Rev. 2011 Jan 19;(1):CD003648. doi: 10.1002/14651858.CD003648.pub3. Update in: Cochrane Database Syst Rev. 2017 Sep 07;9:CD003648. PMID: 21249656.
53. Kuete M, Yuan H, Tchoua Kemayou AL, Songo EA, Yang F, Ma X, Xiong C, Zhang H. Scale up use of family planning services to prevent maternal transmission of HIV among discordant couples: a cross-sectional study within a resource-limited setting. Patient Prefer Adherence. 2016 Oct 3;10:1967-1977. doi: 10.2147/PPA.S105624. PMID: 27757019; PMCID: PMC5055043.
54. Bigna JJ, Noubiap JJ, Kouanfack C, Plottel CS, Koulla-Shiro S. Effect of mobile phone reminders on follow-up medical care of children exposed to or infected with HIV in Cameroon (MORE CARE): a multicentre, single-blind, factorial, randomised controlled trial. Lancet Infect Dis. 2014 Jul;14(7):600-8. doi: 10.1016/S1473-3099(14)70741-8. Epub 2014 Jun 2. PMID: 24932893.
55. Loriette M, Birguel J, Damza R, Ratoua M, Karsikam S, Sobnangou JJ, Aurenche C, Lunel-Fabiani F, Huraux JM. Une expérience de lutte contre l'hépatite B en zone rurale à l'extrême nord du Cameroun [An experience of hepatitis B control in a rural area in Far North Cameroon]. Med Sante Trop. 2015 Oct-Dec;25(4):422-7. French. doi: 10.1684/mst.2015.0507. PMID: 26643767.
56. Bongajum AY, Dufe DM, Tjek PTB, Goon DT, Nkenfou CN, Nwobegahay JM, Mbu R. An assessment of antiretroviral drug initiation to pregnant women of unknown HIV status during labour and delivery in Cameroon. Afr J AIDS Res. 2018 Sep;17(3):265-271. doi: 10.2989/16085906.2018.1515780. Epub 2018 Oct 14. PMID: 30319023.
57. Bain LE, Dierickx K, Hens K. Ethical issues surrounding the provider initiated opt--Out prenatal HIV screening practice in Sub-Saharan Africa: a literature review. BMC Med Ethics. 2015 Oct 24;16(1):73. doi: 10.1186/s12910-015-0068-y. PMID: 26499186; PMCID: PMC4619472.
58. Shey WI, Brocklehurst P, Sterne JA. Vaginal disinfection during labour for reducing the risk of mother-to-child transmission of HIV infection. Cochrane Database Syst Rev. 2002;(3):CD003651. doi: 10.1002/14651858.CD003651. Update in: Cochrane Database Syst Rev. 2005;(4):CD003651. PMID: 12137703.
59. Reinsma K, Nkuoh G, Nshom E. The potential effectiveness of the nutrition improvement program on infant and young child feeding and nutritional status in the Northwest and Southwest regions of Cameroon, Central Africa. BMC Health Serv Res. 2016 Nov 15;16(1):654. doi: 10.1186/s12913-016-1899-z. Erratum in: BMC Health Serv Res. 2017 Feb 24;17 (1):165. PMID: 27846828; PMCID: PMC5109805.
60. Egbe TO, Tazinya RM, Halle-Ekane GE, Egbe EN, Achidi EA. Estimating HIV Incidence during Pregnancy and Knowledge of Prevention of Mother-to-Child Transmission with an Ad Hoc Analysis of Potential Cofactors. J Pregnancy. 2016;2016:7397695. doi: 10.1155/2016/7397695. Epub 2016 Mar 31. PMID: 27127653; PMCID: PMC4830744.
61. Orne-Gliemann J, Tchendjou PT, Miric M, Gadgil M, Butsashvili M, Eboko F, Perez-Then E, Darak S, Kulkarni S, Kamkamidze G, Balestre E, du Loû AD, Dabis F. Couple-oriented prenatal HIV counseling for HIV primary prevention: an acceptability study. BMC Public Health. 2010 Apr 19;10:197. doi: 10.1186/1471-2458-10-197. PMID: 20403152; PMCID: PMC2873579.
62. Etienne L, Locatelli S, Ayouba A, Esteban A, Butel C, Liegeois F, Aghokeng A, Delaporte E, Mpoudi Ngole E, Peeters M. Noninvasive follow-up of simian immunodeficiency virus infection in wild-living nonhabituated western lowland gorillas in Cameroon. J Virol. 2012 Sep;86(18):9760-72. doi: 10.1128/JVI.01186-12. Epub 2012 Jun 27. PMID: 22740419; PMCID: PMC3446613.
63. Chi BH, Tih PM, Zanolini A, Stinson K, Ekouevi DK, Coetzee D, Welty TK, Bweupe M, Shaffer N, Dabis F, Stringer EM, Stringer JS. Implementation and Operational Research: Reconstructing the PMTCT Cascade Using Cross-sectional Household Survey Data: The PEARL Study. J Acquir Immune Defic Syndr. 2015 Sep 1;70(1):e5-9. doi: 10.1097/QAI.0000000000000718. PMID: 26068722; PMCID: PMC4773194.
64. Freeman A, Newman J, Hemingway-Foday J, Iriondo-Perez J, Stolka K, Akam W, Balimba A, Kalenga L, Mbaya M, Mfangam Molu B, Mukumbi H, Niyongabo T, Woelk G, Kiumbu M, Atibu J. Comparison of HIV-positive women with children and without children accessing HIV care and treatment in the IeDEA Central Africa cohort. AIDS Care. 2012;24(6):673-9. doi: 10.1080/09540121.2011.630364. Epub 2011 Nov 22. PMID: 22107066.
65. Orne-Gliemann J, Balestre E, Tchendjou P, Miric M, Darak S, Butsashvili M, Perez-Then E, Eboko F, Plazy M, Kulkarni S, Desgrées du Loû A, Dabis F; Prenahtest ANRS 12127 Study Group. Increasing HIV testing among male partners. AIDS. 2013 Apr 24;27(7):1167-77. doi: 10.1097/QAD.0b013e32835f1d8c. PMID: 23343912.
66. Nakakeeto ON, umaranayake L. The global strategy to eliminate HIV infection in infants and young children: a seven-country assessment of costs and feasibility. AIDS. 2009 May 15;23(8):987-95. doi: 10.1097/qad.0b013e32832a17e9. PMID: 19425224.
67. Muko KN, Tchangwe GK, Ngwa VC, Njoya L. Preventing mother-to-child transmission: factors affecting mothers' choice of feeding--a case study from Cameroon. SAHARA J. 2004 Nov;1(3):132-8. doi: 10.1080/17290376.2004.9724836. PMID: 17601001.
68. Turriziani O, Russo G, Lichtner M, Stano A, Tsague G, Maida P, Vullo V, Antonelli G. Study of the genotypic resistant pattern in HIV-infected women and children from rural west Cameroon. AIDS Res Hum Retroviruses. 2008 Jun;24(6):781-5. doi: 10.1089/aid.2007.0213. PMID: 18507527.
69. Welty TK, Bulterys M, Welty ER, Tih PM, Ndikintum G, Nkuoh G, Nkfusai J, Kayita J, Nkengasong JN, Wilfert CM. Integrating prevention of mother-to-child HIV transmission into routine antenatal care: the key to program expansion in Cameroon. J Acquir Immune Defic Syndr. 2005 Dec 1;40(4):486-93. doi: 10.1097/01.qai.0000163196.36199.89. PMID: 16280706.
70. Ekouevi DK, Stringer E, Coetzee D, Tih P, Creek T, Stinson K, Westfall AO, Welty T, Chintu N, Chi BH, Wilfert C, Shaffer N, Stringer J, Dabis F. Health facility characteristics and their relationship to coverage of PMTCT of HIV services across four African countries: the PEARL study. PLoS One. 2012;7(1):e29823. doi: 10.1371/journal.pone.0029823. Epub 2012 Jan 20. PMID: 22276130; PMCID: PMC3262794.
71. Fru FS, Chiabi A, Nguefack S, Mah E, Takou V, Bogne JB, Lando M, Tchokoteu PF, Mbonda E. Baseline demographic, clinical and immunological profiles of HIV-infected children at the Yaounde Gynaeco-Obstetric and Pediatric hospital, Cameroon. Pan Afr Med J. 2014 Feb 3;17:87. doi: 10.11604/pamj.2014.17.87.3264. PMID: 25452833; PMCID: PMC4247732.
72. Atanga PN, Ndetan HT, Achidi EA, Meriki HD, Hoelscher M, Kroidl A. Retention in care and reasons for discontinuation of lifelong antiretroviral therapy in a cohort of Cameroonian pregnant and breastfeeding HIV-positive women initiating 'Option B+' in the South West Region. Trop Med Int Health. 2017 Feb;22(2):161-170. doi: 10.1111/tmi.12816. Epub 2016 Dec 18. PMID: 27865052.
73. Fokam J, Bellocchi MC, Armenia D, Nanfack AJ, Carioti L, Continenza F, Takou D, Temgoua ES, Tangimpundu C, Torimiro JN, Koki PN, Fokunang CN, Cappelli G, Ndjolo A, Colizzi V, Ceccherini-Silberstein F, Perno CF, Santoro MM. Next-generation sequencing provides an added value in determining drug resistance and viral tropism in Cameroonian HIV-1 vertically infected children. Medicine (Baltimore). 2018 Mar;97(13):e0176. doi: 10.1097/MD.0000000000010176. PMID: 29595649; PMCID: PMC5895385.
74. Wiysonge CS, Shey M, Kongnyuy EJ, Sterne JA, Brocklehurst P. Vitamin A supplementation for reducing the risk of mother-to-child transmission of HIV infection. Cochrane Database Syst Rev. 2011 Jan 19;(1):CD003648. doi: 10.1002/14651858.CD003648.pub3. Update in: Cochrane Database Syst Rev. 2017 Sep 07;9:CD003648. PMID: 21249656.
75. Divaris K, Newman J, Hemingway-Foday J, Akam W, Balimba A, Dusengamungu C, Kalenga L, Mbaya M, Molu BM, Mugisha V, Mukumbi H, Mushingantahe J, Nash D, Niyongabo T, Atibu J, Azinyue I, Kiumbu M, Woelk G. Adult HIV care resources, management practices and patient characteristics in the Phase 1 IeDEA Central Africa cohort. J Int AIDS Soc. 2012 Nov 21;15(2):17422. doi: 10.7448/IAS.15.2.17422. PMID: 23199800; PMCID: PMC3504932.
76. Desclaux A, Alfieri C. Counseling and choosing between infant-feeding options: overall limits and local interpretations by health care providers and women living with HIV in resource-poor countries (Burkina Faso, Cambodia, Cameroon). Soc Sci Med. 2009 Sep;69(6):821-9. doi: 10.1016/j.socscimed.2009.06.007. Epub 2009 Jun 24. PMID: 19559512.
77. Aghokeng AF, Kouanfack C, Eymard-Duvernay S, Butel C, Edoul GE, Laurent C, Koulla-Shiro S, Delaporte E, Mpoudi-Ngole E, Peeters M. Virological outcome and patterns of HIV-1 drug resistance in patients with 36 months' antiretroviral therapy experience in Cameroon. J Int AIDS Soc. 2013 Jan 31;16(1):18004. doi: 10.7448/IAS.16.1.18004. PMID: 23374858; PMCID: PMC3562358.
78. Kfutwah AK, Ngono V, Ngoupo PA, Njouom R. An antiretroviral drug-naïve human immunodeficiency virus-1 infected woman with a persistent non-reactive proviral deoxyribonucleic acid polymerase chain reaction: a case report. J Med Case Rep. 2013 Jun 10;7:152. doi: 10.1186/1752-1947-7-152. PMID: 23759122; PMCID: PMC3689611.
79. Ndawinz JD, Chaix B, Koulla-Shiro S, Delaporte E, Okouda B, Abanda A, Tchomthe S, Mboui E, Costagliola D, Supervie V. Factors associated with late antiretroviral therapy initiation in Cameroon: a representative multilevel analysis. J Antimicrob Chemother. 2013 Jun;68(6):1388-99. doi: 10.1093/jac/dkt011. Epub 2013 Feb 7. PMID: 23391713.
80. Jashi M, Viswanathan R, Ekpini R, Chandan U, Idele P, Luo C, Legins K, Chatterjee A. Informing policy and programme decisions for scaling up the PMTCT and paediatric HIV response through joint technical missions. Health Policy Plan. 2013 Jul;28(4):367-74. doi: 10.1093/heapol/czs067. Epub 2012 Jul 23. PMID: 22826516.
81. Mutarambirwa HD, Kenfack B, Fouogue JT. Term Abdominal Pregnancy Revealed by Amnioperitoneum in Rural Area. Case Rep Obstet Gynecol. 2017;2017:4096783. doi: 10.1155/2017/4096783. Epub 2017 Feb 26. PMID: 28331644; PMCID: PMC5346379.
82. Tita AT, Selwyn BJ, Waller DK, Kapadia AS, Dongmo S. Factors associated with the awareness and practice of evidence-based obstetric care in an African setting. BJOG. 2006 Sep;113(9):1060-6. doi: 10.1111/j.1471-0528.2006.01042.x. PMID: 16956337.
83. Bigna JJ, Noubiap JJ, Plottel CS, Kouanfack C, Koulla-Shiro S. Factors associated with non-adherence to scheduled medical follow-up appointments among Cameroonian children requiring HIV care: a case-control analysis of the usual-care group in the MORE CARE trial. Infect Dis Poverty. 2014 Dec 3;3(1):44. doi: 10.1186/2049-9957-3-44. PMID: 25671122; PMCID: PMC4322435.
84. Menu E, Mognetti B, Moussa M, Nardese V, Tresoldi L, Tscherning C, Mbopi Keou FX, Dubanchet S, Mauclere P, Fenyö EM, Scarlatti G, Barre-Sinoussi F, Chaouat G. Insights into the mechanisms of vertical transmission of HIV-1. BIOMED2 Working Group on the in utero transmission of HIV-1. Early Pregnancy. 1997 Dec;3(4):245-58. PMID: 10086075.
85. Conclusions of a round-table that took place during a seminar on the prevention of TB and HIV transmission in health care settings, Douala, Cameroon, January 1995. Midwifery. 1996 Mar;12(1):39-40. doi: 10.1016/s0266-6138(96)90043-1. PMID: 8715935.
86. Kfutwah AK, Mary JY, Nicola MA, Blaise-Boisseau S, Barré-Sinoussi F, Ayouba A, Menu E. Tumour necrosis factor-alpha stimulates HIV-1 replication in single-cycle infection of human term placental villi fragments in a time, viral dose and envelope dependent manner. Retrovirology. 2006 Jun 23;3:36. doi: 10.1186/1742-4690-3-36. PMID: 16796744; PMCID: PMC1533858.
87. Njom Nlend AE, Same Ekobo C, Moyo ST, Nguetcheng GC, Ngang P, Lyeb S, Meka L, Baane M. Virological profile of pregnant HIV positive women with high levels of CD4 count in low income settings: can viral load help as eligibility criteria for maternal triple ARV prophylaxis (WHO 2010 option B)? Pan Afr Med J. 2011;10:27. doi: 10.4314/pamj.v10i0.72239. Epub 2011 Oct 25. PMID: 22187609; PMCID: PMC3224070.
88. Stringer JS, Stinson K, Tih PM, Giganti MJ, Ekouevi DK, Creek TL, Welty TK, Chi BH, Wilfert CM, Shaffer N, Stringer EM, Dabis F, Coetzee D. Measuring coverage in MNCH: population HIV-free survival among children under two years of age in four African countries. PLoS Med. 2013;10(5):e1001424. doi: 10.1371/journal.pmed.1001424. Epub 2013 May 7. PMID: 23667341; PMCID: PMC3646218.
89. Kfutwah A, Mary JY, Lemen B, Leke R, Rousset D, Barré-Sinoussi F, Nerrienet E, Menu E, Ayouba A; ANRS 1267 study team. Plasmodium falciparum infection significantly impairs placental cytokine profile in HIV infected Cameroonian women. PLoS One. 2009 Dec 2;4(12):e8114. doi: 10.1371/journal.pone.0008114. PMID: 19956547; PMCID: PMC2780732.
90. Egbe TO, Tchente CN, Nkwele GM, Nyemb JE, Barla EM, Belley-Priso E. Cesarean delivery technique among HIV positive women with sub-optimal antenatal care uptake at the Douala General Hospital, Cameroon: case series report. BMC Res Notes. 2017 Jul 26;10(1):332. doi: 10.1186/s13104-017-2639-0. PMID: 28747213; PMCID: PMC5530460.
91. Chaix-Baudier ML, Chappey C, Burgard M, Letourneur F, Igual J, Saragosti S, Rouzioux C. First case of mother-to-infant HIV type 1 group O transmission and evolution of C2V3 sequences in the infected child. French HIV Pediatric Cohort Study Group. AIDS Res Hum Retroviruses. 1998 Jan 1;14(1):15-23. doi: 10.1089/aid.1998.14.15. PMID: 9453247.
92. Skalsky JA, Joller-Jemelka HI, Bianchi L, Knoblauch M. Die Rolle hepatotroper Viren in der Leberpathologie Südwestkameruns [Role of hepatotropic viruses in liver pathology in Southwestern Cameroon]. Schweiz Med Wochenschr Suppl. 1996;79:36S-43S. German. PMID: 8701258.
93. Boeke CE, Joseph J, Wang M, Abate ZM, Atem C, Coulibaly KD, Kebede A, Kiernan B, Kingwara L, Mangwendeza P, Maparo T, Mbaye RN, Mukungunugwa S, Ngugi C, Nzuobontane D, Okomo Assoumou MC, Reta Y, Wambugu B, Rioja MR, Peter T, Doi N, Vojnov L, Khan S, Sacks JA. Point-of-care testing can achieve same-day diagnosis for infants and rapid ART initiation: results from government programmes across six African countries. J Int AIDS Soc. 2021 Mar;24(3):e25677. doi: 10.1002/jia2.25677. PMID: 33745234; PMCID: PMC7981587.
94. Abange WB, Martin C, Nanfack AJ, Yatchou LG, Nusbacher N, Nguedia CA, Kamga HG, Fokam J, Kennedy SP, Ndjolo A, Lozupone C, Nkenfou CN. Alteration of the gut fecal microbiome in children living with HIV on antiretroviral therapy in Yaounde, Cameroon. Sci Rep. 2021 Apr 7;11(1):7666. doi: 10.1038/s41598-021-87368-8. PMID: 33828220; PMCID: PMC8027858.
95. Fomulu, JN & Nana, PN & Nkwabong, Elie & Wamba, FT & Foumane, P & Mbu, R & Tebeu, Pierre Marie. (2009). Efficacy of highly active triple antiretroviral therapy in preventing mother-to-child HIV transmission in the university teaching hospitals in Yaounde, Cameroom. Clinics in Mother and Child Health. 6.
96. Awuba, Jude & Macassa, Gloria. (2008). HIV/AIDS in Cameroon: Rising gender issues in policy-making matters. African Journal of Health Sciences (ISSN: 1022-9272) Vol 14 Num 3-4. 14. 10.4314/ajhs.v14i3.30857.
97. Amenu, Desalegn. (2015). Malaria and HIV/AIDS Co-infections. Int. J. Curr. Res. Med. Sci. 1. 16-22.
98. Tanjong, Rebecca & Teyim, Pride & Kamga, Henri Lucien & Neba, Edwin & Nkuo-Akenji, Theresia. (2016). Sero-prevalence of Human Immunodeficiency Virus and hepatitis viruses and their correlation with CD4 T-cell lymphocyte counts in pregnant women in the Buea Health District of Cameroon. International Journal of Biological and Chemical Sciences. 10. 219. 10.4314/ijbcs.v10i1.17.
99. Bwana, Veneranda & Frimpong, Christiana & Simulundu, Edgar & Mfinanga, Sayoki & Mboera, Leonard & Michelo, Charles. (2016). Accessibility of services for early infant diagnosis of Human Immunodeficiency Virus in sub-Saharan Africa: a systematic review. Tanzania Journal of Health Research. 18. 10.4314/thrb.v18i3.9.
100. Bongajum, Anastasia & Dufe, Divine & Tjek, Paul & Ter, Goon & Nkenfou, Céline & Nwobegahay, Julius & Mbu, Robinson. (2018). An assessment of antiretroviral drug initiation to pregnant women of unknown HIV status during labour and delivery in Cameroon. African Journal of AIDS Research. 17. 1-7. 10.2989/16085906.2018.1515780.
101. Hamela, Gloria & Kabondo, Charity & Tembo, Tapiwa & Kamanga, Esmie & Mofolo, Innocent & Bulla, Bertha & Sellers, Christopher & Nakanga, R.C. & Lee, Clara & Martinson, Francis & Hoffman, Irving & van der Horst, Charlie & Hosseinipour, Mina. (2014). Evaluating the Benefits of Incorporating Traditional Birth Attendants in HIV Prevention of Mother to Child Transmission Service Delivery in Lilongwe, Malawi. African journal of reproductive health. 18. 27-34.
102. Temitope, Ashipa & Ofili, Antoinette & JUE, Onakewhor & Adejumo, Olusola. (2017). Health Workers' Knowledge of Preventing Mother-To-Child Transmission of HIV in Benin City, Edo State, Nigeria. Journal of Community Medicine and Primary Health Care. 29. 1 -10.
103. Disclosure of illness status to children and adolescents with HIV infection. American Academy of Pediatrics Committee on Pediatrics AIDS. Pediatrics. 1999 Jan;103(1):164-6. doi: 10.1542/peds.103.1.164. PMID: 9917458.
104. Francisca, Monebenimp & Nga-Essono, Dorothee & Bissek, Anne-Cecile & Chelo, David & Tetanye, Ekoe. (2011). HIV exposure and related newborn morbidity and mortality in the University Teaching Hospital of Yaoundé, Cameroon. The Pan African medical journal. 8. 43. 10.4314/pamj.v8i1.71160.
105. Manji K P, Manji M P and Sherifi F G. (2011). The use of Anti-Retroviral Drugs in the prevention of Mother To Child Transmission (PMTCT) of HIV Review Paper. Tanzania Medical Journal. 25(2).
106. Ugoeze, K. and C. Emenike. “Comparison of performance of prevention of mother-to-child transmission (PMTCT) of HIV/AIDS cascade between public and private health facilities in a community PMTCT implementation in north central Nigeria.” Journal of Pharmaceutical and Allied Sciences 13 (2016): 2373-2381.
107. Teclebirhan, T & Berhane, Araia & Mufunda, Jacob & Gebremichael, A. (2010). Prevention of mother to child transmission of HIV/AIDS in Eritrea: the Eritrean experience. Journal of the Eritrean Medical Association. 4. 10.4314/jema.v4i1.52130.
108. Teasdale, Chloe & Besser, Mitchell. (2008). Enhancing PMTCT programmes through psychosocial support and empowerment of women: The Mothers2mothers model of care. S Afr J HIV Med. 9.
109. Djuidje Ngounoue, Marceline & Djikeng, Appolinaire & Spiro, David & Mbangue, Madeleine & Kuate, Honoré & Moundipa, Paul & Kaptue, Lazare. (2015). Human Immunodeficiency Virus and Hepatitis C Virus Co-infection in Cameroon: Investigation of the Genetic Diversity and Virulent Circulating Strains. JOURNAL OF THE CAMEROON ACADEMY OF SCIENCES.
110. Andreas, Chiabi & Lebela, Jacqueline & Kobela, Marie & Mbuagbaw, Lawrence & Obama, Marie & Ekoe, Tetanye. (2012). The frequency and magnitude of growth failure in a group of HIV-infected children in Cameroon. The Pan African medical journal. 11. 15. 10.11604/pamj.2012.11.15.1297.
111. Isah HO, Dakum P. Institutional Profile of PHC Facilities in Nigeria and its Implication for PMTCT Scale-Up and Decentralization of HIV and AIDS Services: Nasarawa State as a Case Study. Journal of Pharmaceutical and Allied Sciences; 11(1).
112. Mkoma, George. (2012). Improving Child survival through enhancing Prevention of Mother to Child Transmission of HIV. Dar Es Salaam Medical Students' Journal. 19. 10.4314/dmsj.v19i1.6.
113. Nsojo, Anthony & Aboud, Said & Lyamuya, Elna. (2010). Comparative evaluation of Amplicor HIV-1 DNA test, version 1.5, by manual and automated DNA extraction methods using venous blood and dried blood spots for HIV-1 DNA PCR testing. Tanzania journal of health research. 12. 229-35. 10.4314/thrb.v12i4.58621.
114. Igumbor, Jude & Pengpid, Supa & Obi, Chibuzo. (2006). Effect of exposure to clinic-based health education interventions on behavioural intention to prevent mother-to-child transmission of HIV infection. SAHARA J : journal of Social Aspects of HIV/AIDS Research Alliance / SAHARA , Human Sciences Research Council. 3. 394-402. 10.1080/17290376.2006.9724865.
115. Otieno, Allan & Kagira, John & Otieno, A & Karanja, Simon. (2018). KNOWLEDGE AND ATTITUDE AS DETERMINANT FACTORS IN HIV CARE AMONG PREGNANT WOMEN IN RACHUONYO NORTH, HOMA-BAY COUNTY, KENYA. East African medical journal. 95.
116. R Nduati, J Oyieke, R Mbayaki, R Musyoka, R Kamau, R Ayisi, J Deya, D Mbori-Ngacha. (2019). Roll-out of prevention of mother-to-child transmission of Human Immunodeficiency Virus in rural Kenya. East African Medical Journal; 96(5).
117. Lejeng, Rose Opiyo Okoyo and Joyce Olenja. Mothers’ knowledge of mother-to-child transmission of HIV and infant feeding practices in Juba, South Sudan. South Sudan Medical Journal 2020; 13(3):79-85.
118. Negash TG, Ehlers VJ. Women's utilisation of prevention of mother-to-child transmission of human immunodeficiency virus services in Addis Ababa, Ethiopia. Health SA. 2018 Aug 27;23:1145. doi: 10.4102/hsag.v23i0.1145. PMID: 31934391; PMCID: PMC6917383
119. King, Rachel & Matovu, Joyce & Rujumba, Joseph & Wavamunno, Priscilla & Amone, Alexander & Gabagaya, Grace & Fowler, Mary & Homsy, Jaco & Seeley, Janet & Musoke, Philippa. (2020). PMTCT Option B+ 2012 to 2018 — Taking stock: barriers and strategies to improve adherence to Option B+ in urban and rural Uganda. African Journal of AIDS Research. 19. 135-146. 10.2989/16085906.2020.1760325.
120. Samuels, EON & Isah, Aliyu & Offiong, Richard & Ekele, Bissallah. (2014). Foeto-maternal outcome of HIV-positive pregnant women on Highly Active Antiretroviral Therapy. International Journal of Medicine and Biomedical Research. 3. 202-208. 10.14194/ijmbr.3.3.8.
121. B Douaguibe, K Fiagnon, T Bassowa, D Ajavon, A.S. Aboubakari, K Akpadza. (2017). Suivi de la femme enceinte seropositive au VIH a l'Hopital de be a Lome. Journal de la recherche scientifique de l’universite de Lome. 19(4).
122. KD Azoumah, AS Aboubakari, KT Nabroulaba, YD Atakouma, B Bakonde, K Tatagan-Agbi. (2012). Prevention de la transmission mere enfant du VIH/SIDA au Chu de Kara (Togo) : Bilan de l’evolution sur quatre ans d’activite. Journal de la recherche scientifique de l’universite de Lome; 14(1).
123. A J W Otieno, John Kagira. (2017). Socio-cultural Factors Influencing Utilization of Prevention-of-Mother-to-Child-Transmission of HIV Strategies among Women Attending Antenatal Care Clinics in Rachuonyo North Sub-County-Homa-Bay County. East African medical journal 94(11).
124. Akani, CI & Erhabor, Osaro & Opurum, Hamilton & Oa, Ejele & Nwauche, Chijioke. (2006). HIV sero-discordance among Nigerian couples: challenges and controversies. Nigerian Medical Practitioner. 48. 10.4314/nmp.v48i3.28766.
125. KATLAMA, C. (1989). Aspects epidemiologiques, cliniques et therapeutiques de l'infection a VIH. Medecine Et Maladies Infectieuses - MED MAL INFEC. 19. 234-240. 10.1016/S0399-077X(89)80081-2.
126. O.E. Takassi, D.A.E. Akolly, K.M. Guedenon, Y.D. Atakouma. (2020). Partage du statut serologique au sein des couples serodiscordants dans un protocole de PTME en pediatrie au CHU Sylvanus Olympio (Togo). Journal de la recherche scientifique de l’universite de Lome ;22(1-2).
127. King R, Matovu JN, Rujumba J, Wavamunno P, Amone A, Gabagaya G, Fowler MG, Homsy J, Seeley J, Musoke P. PMTCT Option B+ 2012 to 2018 - Taking stock: barriers and strategies to improve adherence to Option B+ in urban and rural Uganda. Afr J AIDS Res. 2020 Jul;19(2):135-146. doi: 10.2989/16085906.2020.1760325. PMID: 32780677.
128. HO Isah, P Dakum. Institutional Profile of PHC Facilities in Nigeria and its Implication for PMTCT Scale-Up and Decentralization of HIV and AIDS Services: Nasarawa State as a Case Study. (2014). Journal of Pharmaceutical and Allied Sciences; 11(1).
129. KE Djadou, KD Azoumah, KS Koffi, K Lawson-Evi, D Vinyo, K Tatagan-Agbi. (2012). Connaissances, attitudes et pratiques en focus group des clients des sites de prévention de la transmission du VIH de la mère à son enfant au Togo en 2010. Journal de la Recherche Scientifique de l’Université de Lomé ; 14(1).
130. R, J Oyieke, R Mbayaki, R Musyoka, R Kamau, R Ayisi, J Deya, D Mbori-Ngacha. (2019). Roll-out of prevention of mother-to-child transmission of Human Immunodeficiency Virus in rural Kenya. East African Medical Journal; 96(5).
131. Nguhiu, Purity & African, East. (2017). THE ROLE OF MATERNAL, PSYCHOSOCIAL AND SOCIAL-CULTURAL FACTORS IN HIV-EXPOSED INFANTS' SERVICE UPTAKE. East African medical journal. 94. 1010- 1027.
132. CHIABI A.; IMUNBOEH P. 1; YANG XIAO YAN 1; BEYEME-OWONO M. (2006). DISCLOSURE OF HIV ILLNESS STATUS TO CHILDREN AND ADOLESCENTS: When, how and by who? Clin Mother Child Healt; 3(1): 509-512.
133. Esemu LF, Yuosembom EK, Fang R, Rasay S, Fodjo BAY, Nguasong JT, Kidima W, Ekali GL, Chen JJ, Ndhlovu L, Bigoga JD, Taylor DW, Leke RGF, Babakhanyan A. Impact of HIV-1 infection on the IGF-1 axis and angiogenic factors in pregnant Cameroonian women receiving antiretroviral therapy. PLoS One. 2019 May 1;14(5):e0215825. doi: 10.1371/journal.pone.0215825.
134. Penda CI, Moukoko CEE, Koum DK, Fokam J, Meyong CAZ, Talla S, Ndombo PK. Feasibility and utility of active case finding of HIV-infected children and adolescents by provider-initiated testing and counselling: evidence from the Laquintinie hospital in Douala, Cameroon. BMC Pediatr. 2018 Aug 3;18(1):259. doi: 10.1186/s12887-018-1235-3.
135. Yumo HA, Ndenkeh JN Jr, Sieleunou I, Nsame DN, Kuwoh PB, Beissner M, Loscher T, Kuaban C. Human immunodeficiency virus case detection and antiretroviral therapy enrollment among children below and above 18 months old: A comparative analysis from Cameroon. Medicine (Baltimore). 2021 Apr 30;100(17):e25510. doi: 10.1097/MD.0000000000025510.
136. Mkoma, George. (2012). Improving Child survival through enhancing Prevention of Mother to Child Transmission of HIV. Dar Es Salaam Medical Students' Journal. 19. 10.4314/dmsj.v19i1.6.
137. Aregbesola, Oluwabusayo & Adeoye, Ikeola. (2018). Self-efficacy and antiretroviral therapy adherence among HIV positive pregnant women in South-West Nigeria: a mixed methods study. Tanzania Journal of Health Research. 20. 10.4314/thrb.v20i4.x.
138. Negash, Tefera & Ehlers, Valerie. (2018). Women’s utilisation of prevention of mother-to-child transmission of human immunodeficiency virus services in Addis Ababa, Ethiopia. Health SA Gesondheid. 23. 10.4102/hsag.v23i0.1145.
139. Irinyenikan, Theresa. (2019). Knowledge and practice of HIV testing for PMTCT among antenatal clinic attendees. Tropical Journal of Obstetrics and Gynaecology. 36. 232. 10.4103/TJOG.TJOG_96_18.
